# Multiomics reveals multilevel control of renal and systemic metabolism by the renal tubular circadian clock

**DOI:** 10.1172/JCI167133

**Published:** 2023-04-17

**Authors:** Yohan Bignon, Leonore Wigger, Camille Ansermet, Benjamin D. Weger, Sylviane Lagarrigue, Gabriel Centeno, Fanny Durussel, Lou Götz, Mark Ibberson, Sylvain Pradervand, Manfredo Quadroni, Meltem Weger, Francesca Amati, Frédéric Gachon, Dmitri Firsov

**Affiliations:** 1 Department of Biomedical Sciences, University of Lausanne, Lausanne, Switzerland.; 2 Vital-IT, Swiss Institute of Bioinformatics, Lausanne, Switzerland.; 3 Genomic Technologies Facility, University of Lausanne, Lausanne, Switzerland.; 4 Institute for Molecular Bioscience, The University of Queensland, Brisbane, Australia.; 5 Protein Analysis Facility, University of Lausanne, Lausanne, Switzerland.

**Keywords:** Nephrology, Bioenergetics, Bioinformatics, Fatty acid oxidation

## Abstract

Circadian rhythmicity in renal function suggests rhythmic adaptations in renal metabolism. To decipher the role of the circadian clock in renal metabolism, we studied diurnal changes in renal metabolic pathways using integrated transcriptomic, proteomic, and metabolomic analysis performed on control mice and mice with an inducible deletion of the circadian clock regulator *Bmal1* in the renal tubule (cKOt). With this unique resource, we demonstrated that approximately 30% of RNAs, approximately 20% of proteins, and approximately 20% of metabolites are rhythmic in the kidneys of control mice. Several key metabolic pathways, including NAD^+^ biosynthesis, fatty acid transport, carnitine shuttle, and β-oxidation, displayed impairments in kidneys of cKOt mice, resulting in perturbed mitochondrial activity. Carnitine reabsorption from primary urine was one of the most affected processes with an approximately 50% reduction in plasma carnitine levels and a parallel systemic decrease in tissue carnitine content. This suggests that the circadian clock in the renal tubule controls both kidney and systemic physiology.

## Graphical abstract

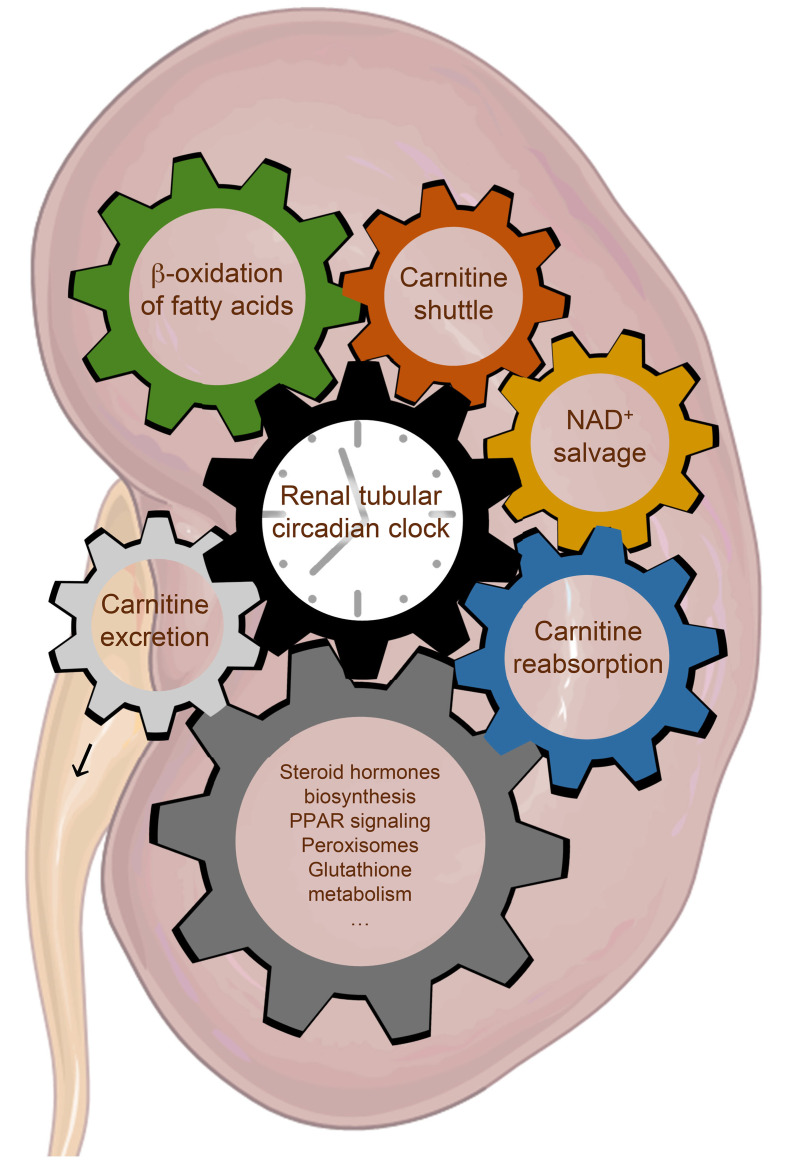

## Introduction

Most basic physiological functions, including the process of urine formation in kidneys, exhibit substantial daily oscillations orchestrated by the circadian system. This circadian system allows organisms to anticipate and prepare for changing functional demands throughout the day and night cycles. In mammals, the circadian timing system is hierarchically organized with a central clock located in the suprachiasmatic nucleus (SCN) of the hypothalamus that coordinates the circadian clocks in peripheral tissues (peripheral clocks) to keep phase coherence between the different tissue clocks (1). At the molecular level, the circadian clock is based on series of transcriptional and translational feedback loops that drive rhythmic expression of numerous genes in an organ-specific manner (2).

Daily rhythms in specific renal functions are generated and maintained by a variety of factors, including the intrinsic renal circadian clock and the rhythmic systemic stimuli orchestrated by the central circadian clock, such as circulating metabolites or hormones (reviewed in refs. 3, 4). In humans, disruption of circadian rhythms due to shift work or sleep disorders are associated with a decreased glomerular filtration rate (GFR) (5), increased risk of chronic kidney disease (CKD) (6), polyuria, and nocturia (7). Systemic perturbation of the circadian clock in different transgenic mouse models resulted in a partial loss of blood pressure control (8), substantial changes in the circadian pattern of urinary water, sodium and potassium excretion (9), and accelerated progression of CKD (10). Conditional deletion of the circadian clock gene *Bmal1* (brain and muscle ARNT-like 1, also known as *Arntl*) in glomerular podocytes caused disruption of the circadian rhythm in GFR and parallel alterations in the circadian patterns of plasma aldosterone levels and urinary excretion of creatinine, sodium, potassium, and water (11). Inactivation of *Bmal1* in the renal tubule does not lead to an overt phenotype in unstressed conditions, except a reduction (of approximately 20%) in kidney weight and kidney weight–to–body weight ratio (12). However, in a model of type I diabetes, these mice displayed exacerbated hyperglycemia caused by the enhancement of the gluconeogenic pathway in the kidney (13). These and other results gave rise to the hypothesis of a second-hit role of the renal tubular circadian clock in the development of kidney and/or systemic diseases. In the absence of other hits, the dysfunction of renal tubular clocks does not lead to an overt phenotype. However, in stress or disease situations, the disorganization of renal metabolic pathways and/or transport processes along the renal tubule may become critical. Moreover, a large body of literature exists on the bidirectional relationship between CKD and the circadian clock system, where CKD causes multiorgan or whole-body chronodisruption and the dysfunction of the circadian clock or perturbation of circadian rhythms aggravate CKD and its complications (4).

Urine formation in kidneys involves 3 main processes: glomerular filtration, tubular reabsorption, and tubular secretion. Evidence suggests that tubular reabsorption/secretion varies markedly throughout the day in parallel with the circadian fluctuations in GFR, renal blood flow (RBF), and plasma concentration of some solutes. For instance, GFR displays daily fluctuations with the acrophase, or circadian peak time, occurring in the active phase and amplitude, or the difference between circadian peak and trough, ranging from 20%–50%, depending on species and experimental conditions (14–18). For water and most solutes, this implies parallel rhythmic changes in their filtered loads. Daily oscillations in filtered loads impose, in turn, rhythmic fluctuations in tubular transport processes. Considering that tubular transport is the primary determinant of kidney energy expenditure, this suggests a requirement for daily adjustments in energy production by renal tubular cells. Moreover, daily oscillations in RBF (19) generate substantial fluctuations in kidney tissue oxygenation (20) and metabolic substrate availability, thereby suggesting rhythmic changes in renal metabolic pathways. While the rhythmic regulation of main tubular transporters has been addressed in several studies (21–25), the role of the circadian clock in the adjustment of renal metabolism throughout the day remains largely unknown.

Here, we studied the role of intrinsic renal circadian clocks in the control of renal metabolism. To address this question, we performed an integrated time-resolved analysis of the renal transcriptome and proteome in parallel with the renal and plasma metabolomes in control mice and mice with an induced deletion of *Bmal1* in the renal tubule. Of note, the kidney is composed of several dozen highly differentiated cell types characterized by both overlapping and distinct metabolic programs. For instance, metabolism of the proximal tubule differs from the rest of the nephron by the presence of a high-capacity gluconeogenic pathway and an incapacity to use glucose as metabolic fuel. In contrast, β-oxidation of fatty acids (FAs) is an important source of energy production in all tubular segments, but especially in the proximal tubule. Thus, circadian regulation of metabolic pathways may be different in different parts and/or cell types of the renal tubule.

Our study showed that the intrinsic circadian clock in the renal tubule exerted control over renal metabolism by regulating several key metabolic pathways, including carnitine shuttle, β-oxidation of FAs, and nicotinamide (NAM) adenine dinucleotide (NAD^+^) biosynthesis. As a result, kidney mitochondrial activity was affected, leading unexpectedly to a de novo rhythm in cKOt, suggesting a disruption of the renal energy homeostasis. Moreover, we revealed the critical involvement of the renal circadian clock in the systemic control of tissue carnitine levels. Altogether, the data sets generated here (https://bix.unil.ch/circadian-kidney/) provide a unique resource for the understanding of rhythmic kidney physiology.

## Results

### Effect of induced Bmal1 deletion in the renal tubule on the kidney rhythmic transcriptome.

**Figure 1:**
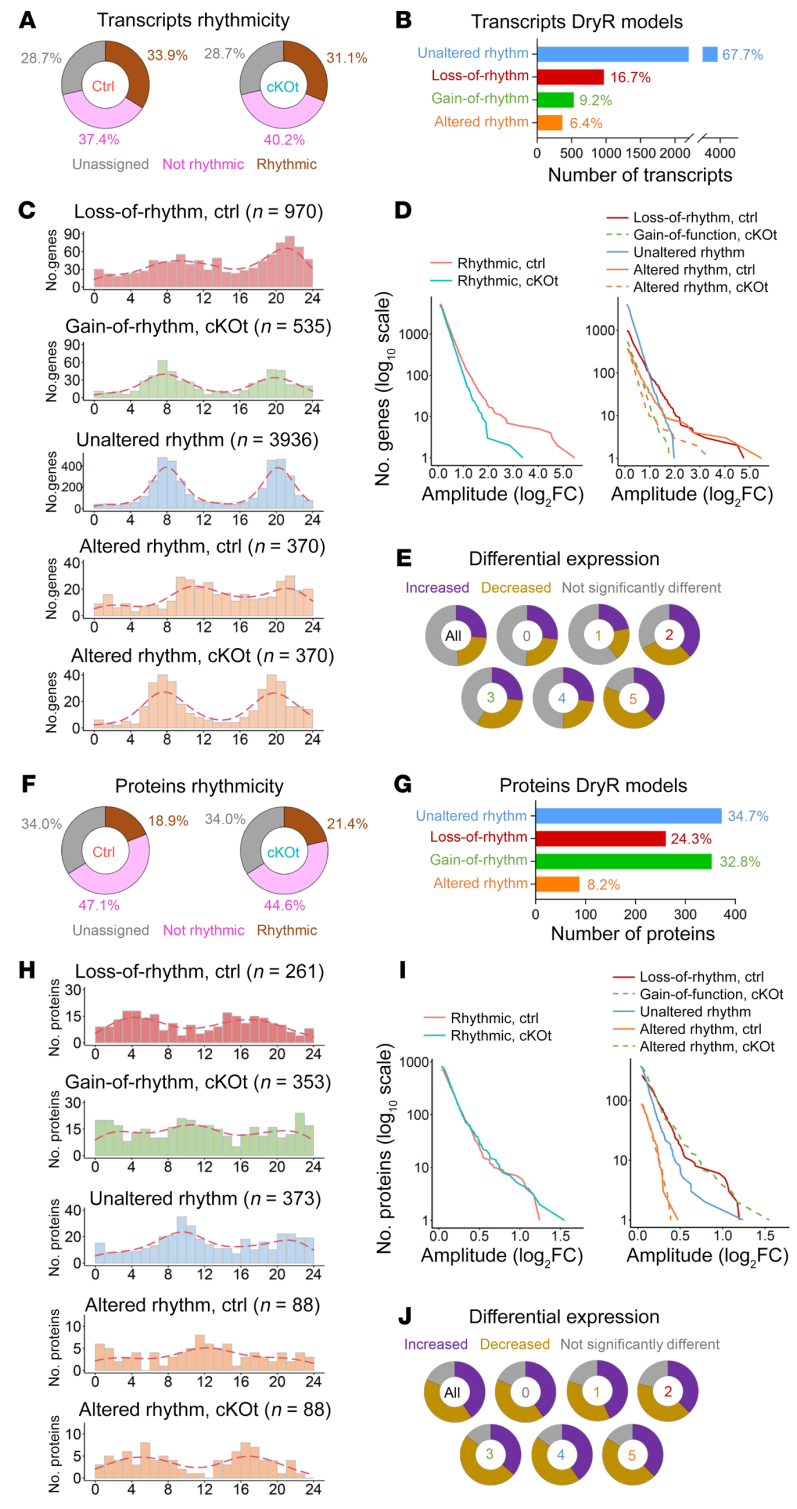
Alterations of renal transcriptome and renal proteome in cKOt mice. (**A**) Donut charts showing the percentage of rhythmic, nonrhythmic and unassigned renal transcripts in control (Ctrl) and cKOt mice. (**B**) Histogram showing the number and percentage of renal rhythmic transcripts assigned to *dryR* rhythmicity models. (**C**) Histogram showing the acrophase distribution of renal transcripts assigned to *dryR* rhythmicity models 2, 3, 4, and 5. Red dashed lines: kernel density estimates. (**D**) Cumulative number of renal transcripts assigned in the indicated rhythmicity pattern in function of amplitude. (**E**) Donut charts showing the proportion of renal transcripts displaying a differential mean expression according to limma R package (72) in Ctrl versus cKOt mice for each *dryR* rhythmicity model. (**F**) Donut charts showing the percentage of rhythmic, not rhythmic, and unassigned renal proteins in Ctrl and cKOt mice. (**G**) Histogram showing the number and percentage of renal rhythmic proteins assigned to dryR rhythmicity models. (**H**) Histogram showing the acrophase distribution of renal proteins assigned to *dryR* rhythmicity models 2, 3, 4, and 5. Red dashed lines: kernel density estimates. (**I**) Cumulative number of renal proteins assigned in the indicated rhythmicity pattern in function of amplitude. (**J**) Donut charts showing the proportion of renal proteins displaying a differential mean expression according to limma R package in control (Ctrl) versus cKOt mice for each *dryR* rhythmicity model.

Inactivation of *Bmal1* in the renal tubule was induced by 2-week treatment with doxycycline (DOX; 2 mg/mL in drinking water) of 8-week-old *Bmal1^lox/lox^*/*Pax8*-*rtTA*/LC1-Cre male mice (hereafter referred to as cKOt mice) (12, 26). Their littermate controls (*Bmal1^lox/lox^* mice; hereafter referred to as control mice) received the same DOX treatment. Kidneys and plasma were collected 1 month after the end of DOX treatment from mice maintained under a standard 12 hour light/12 hour dark cycle with ad libitum feeding (sampled every 4 hours over a 24 hour cycle; 5 independent replicates). We subsequently investigated temporal gene expression in the kidney using RNA-Seq and performed a differential rhythmicity analysis using *dryR* (27) (Supplemental Table 1; supplemental material available online with this article; https://doi.org/10.1172/JCI167133DS1). This analysis is based on multiple harmonic linear regression with a subsequent model selection approach that assigned transcripts to 5 different models according to their rhythmic or nonrhythmic expression pattern in control and cKOt mice (Supplemental Figure 1A). Transcripts assigned to model 1 (nonrhythmic) did not exhibit rhythmicity in both conditions. Model 2 (loss-of-rhythm) comprised transcripts that lost their rhythmic expression pattern in cKOt mice; model 3 (gain-of-rhythm) represented transcripts that gained rhythmicity in cKOt mice; and transcripts that exhibited unaltered rhythms in both genotypes were assigned to model 4 (unaltered rhythm). If the rhythms were altered in acrophase and/or amplitude, transcripts were classified as model 5 (altered rhythm). Transcripts that could not be clearly assigned to one of these models were named “unassigned” (model 0) (Supplemental Figure 1A). Of all detected transcripts, 33.9% (sum of models 2, 4 and 5) and 31.1% (sum of models 3, 4 and 5) were rhythmic in kidneys of control and cKOt mice, respectively (Figure 1A). While the majority (67.7%) of rhythmic transcripts in at least 1 genotype demonstrated unaltered rhythm (system-driven transcripts), 16.7% of transcripts lost their rhythmicity in cKOt mice (BMAL1-driven transcripts), 9.2% gained rhythmicity (transcripts that became rhythmic after the deletion of *Bmal1*), and 6.4% exhibited altered rhythm (BMAL1-modulated transcripts) (Figure 1B and Supplemental Figure 1B). Rhythmic transcripts that lost rhythmicity in cKOt mice (model 2) did not present clear phase enrichment but did show higher amplitudes (Figure 1, C and D), as expected for direct BMAL1 target genes in clock-deprived animals (27). Transcripts from models 3 and 4 exhibited a bimodal distribution of phases and lower amplitudes (Figure 1, C and D), suggesting that they were likely driven by systemic signals. Transcripts with altered rhythm in cKOt mice (model 5) demonstrated a bimodal distribution only in cKOt mice, while the phase was not biased in controls, which was expected for genes regulated by both the circadian clock and systemic signals (27). The phase of these genes was shifted by about –4 or +4 hours and also presented a lower amplitude in cKOt mice than in control mice (Figure 1, C and D and Supplemental Figure 1C). As shown in Figure 1E, a substantial number of transcripts (more than 50%) from models 2, 3, 4, and 5 also displayed a differential mean expression. The differential mean expression was even higher among transcripts with differential rhythmicity (models 2, 3, and 5) suggesting a link between rhythmicity and differential expression (Figure 1E) (27).

### Effect of induced Bmal1 deletion in the renal tubule on the kidney rhythmic proteome.

To decipher the relationship between the rhythmic transcriptome and proteome, we performed, in parallel, a proteomic analysis of kidneys from control and cKOt mice. A total of 3,809 proteins were quantified in both control and cKOt kidney samples (Supplemental Table 2). *dryR* analysis revealed 18.9% and 21.4% of rhythmic proteins in kidneys of control and cKOt mice, respectively (Figure 1F). Among proteins rhythmic in at least 1 genotype, a surprisingly high number (32.8%) gained rhythmicity in the kidneys of cKOt mice, while only 24.3% proteins lost their rhythmicity, 8.2% had altered rhythmicity, and 34.7% showed unaltered rhythm (Figure 1G). Interestingly, only proteins in model 4 (unaltered rhythm) showed a clear bimodal phase distribution as expected for system-driven proteins. Proteins in other models did not display clear biased distribution of phases, likely because of the relatively low number of rhythmic proteins compared with mRNAs (Figures 1H and Supplemental Figure 1D). There was no clear tendency for phase-shift distribution for proteins from model 5 (Supplemental Figure 1E). Proteins that gained or lost rhythmicity (models 2 and 3) showed higher amplitudes compared with the other groups (Figure 1I). While this was expected for model 2 proteins that are likely directly regulated by BMAL1, this was more surprising for proteins that gained rhythmicity (model 3). This suggested that the deletion of *Bmal1* generated compensatory mechanisms that induced the rhythmicity of new proteins in response to a potential modification of renal tubular functions.

Strikingly, a high number of proteins — more than 80% — from all models exhibited differential mean expression between control and cKOt mice (Figure 1J), demonstrating the substantial effect of *Bmal1* deletion on the renal proteome.

### Relationship between the rhythmic transcriptome and the proteome in kidneys of control and cKOt mice.

**Figure 2:**
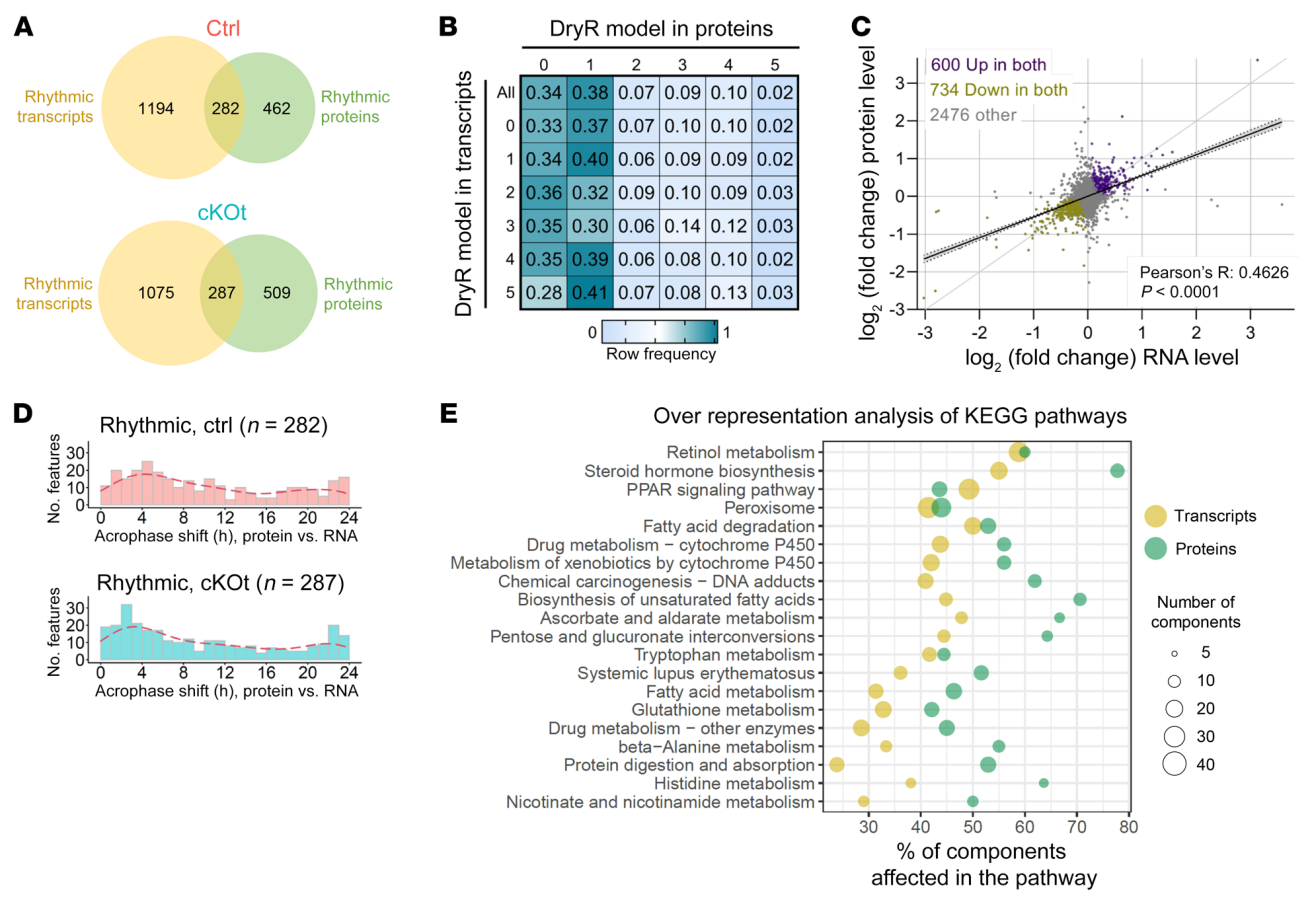
Comparison of changes observed in renal transcriptome and proteome. (**A**) Venn diagram showing the number of rhythmic transcripts and proteins among all detected pairs in control (Ctrl) and cKOt mice. (**B**) Table showing the frequency of transcripts and proteins pairs falling in the same *dryR* rhythmicity model. (**C**) Scatter plot and regression line with 95% confidence intervals of log_2_ fold changes in mean expression between Ctrl and cKOt mice at transcriptional (x axis) and protein (y axis) levels. (**D**) Histogram showing the distribution of the acrophase shift between transcripts and proteins in Ctrl (upper panel) or cKOt (lower panel) mice. Red dashed lines: kernel density estimates. (**E**) Scatter plot of all KEGG metabolic pathways significantly altered (*P*_adj_ < 0.25) in both transcriptomic and proteomic renal data sets. Results are based on over representation analyses (ORA) of transcripts or proteins showing a significantly altered mean expression (*P*_adj_ < 0.05 obtained with limma R package) with an absolute fold change > 1.2. The size of each dot depends on the number of transcripts or proteins, or components, of the pathway significantly affected in cKOt mice. Pathways are sorted from the lower to the higher value obtained by multiplication of *P*_adj_ of both data sets.

Global pairwise comparison of rhythmic mRNA and proteins revealed that only approximately 20% of rhythmic mRNAs encoded rhythmic proteins in the kidneys of control and cKOt mice, while only approximately 37% of rhythmic proteins were encoded by rhythmic mRNAs (Figure 2A). This is consistent with previous results that showed that most rhythmic mRNAs encode nonrhythmic proteins and that most rhythmic proteins are regulated at the posttranscriptional levels (28–31). These data were confirmed by a weak correlation in model-to-model comparisons of rhythmicity patterns between mRNAs and proteins that they encode (Figure 2B). However, a strong correlation was found between fold-changes in mean expression levels of mRNA and protein pairs in control versus cKOt mice (Figure 2C, Pearson’s R correlation coefficient: 0.4626: *P* value<0.0001). A comparison of phase distributions revealed a tendency in both control and cKOt mice for a phase delay of approximately 2-to-6 hours between mRNA and protein expression likely explained by the time of accumulation of proteins after the peak of mRNA synthesis (Figure 2D) (28). Pathway analysis based on over representation analysis (ORA) of mRNAs or proteins exhibiting differential mean expression between control and cKOt mice revealed a substantially similar set of molecular pathways modified on both mRNA and protein levels. Among 30 altered protein pathways, as many as 20 were already affected at the mRNA level (Figure 2E and Supplemental Tables 3 and 4). Notably, xenobiotic detoxification; FA metabolism; proliferator-activated receptor agonist (PPAR) signaling; peroxisomal function; and nicotinate, NAM, and tryptophan metabolism pathways were commonly affected.

### Consequences of Bmal1 deletion in the renal tubule on kidney and plasma metabolomes.

**Figure 3:**
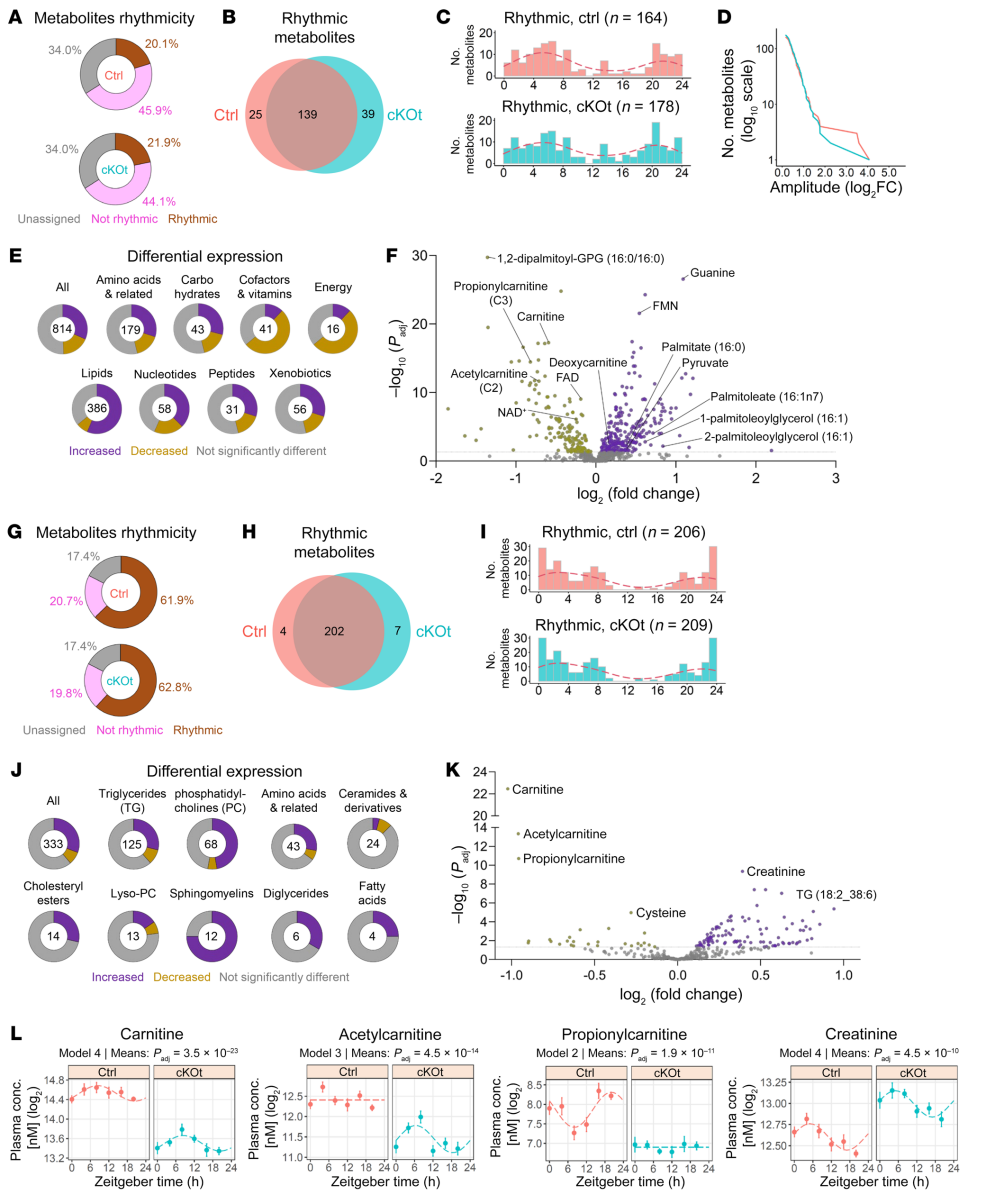
Alterations of renal and plasma metabolomes in cKOt mice. (**A**) Donut charts showing the percentage of rhythmic, nonrhythmic, and unassigned renal metabolites in control (Ctrl) and cKOt mice. (**B**) Venn diagram showing the number of rhythmic metabolites in kidneys of Ctrl and cKOt mice. (**C**) Histogram showing the acrophase distribution of rhythmic metabolites in kidneys of Ctrl and cKOt mice. Red dashed lines: kernel density estimates. (**D**) Cumulative number of rhythmic metabolites in Ctrl and cKOt mice in function of amplitude. (**E**) Donut charts showing the proportion of renal metabolites showing an increased, decreased, or not significantly altered mean level according to limma R package in cKOt versus Ctrl mice for each class of metabolites. (**F**) Volcano plot depicting metabolites significantly (*P*_adj_ < 0.05) more abundant (purple dots) or less abundant (yellow dots) in kidneys of cKOt mice compared with Ctrl mice. (**G**) Donut charts showing the percentage of rhythmic, nonrhythmic, and unassigned plasma metabolites in Ctrl and cKOt mice. (**H**) Venn diagram showing the number of rhythmic metabolites in plasma of Ctrl and cKOt mice. (**I**) Histogram showing the acrophase distribution of rhythmic metabolites in plasma samples of Ctrl and cKOt mice. Red dashed lines: kernel density estimates. (**J**) Volcano plot depicting metabolites significantly (*P*_adj_ < 0.05) more abundant (purple dots) or less abundant (yellow dots) in the plasma of cKOt mice compared with Ctrl mice. (**K**) Donut charts showing the proportion of plasma metabolites showing an increased, decreased, or not significantly altered mean level according to limma R package in cKOt versus Ctrl mice for each class of metabolite. (**L**) Temporal plots showing the plasma concentration, *dryR* rhythmicity model, and limma R package result of mean expression comparison between Ctrl and cKOt mice for carnitine, acetylcarnitine, propionylcarnitine, and creatinine in plasma metabolomes of Ctrl and cKOt mice.

Out of 814 metabolites quantified in the kidneys of control and cKOt mice, only approximately 20% were rhythmic (Figure 3A and Supplemental Table 5). While a majority of metabolites were rhythmic in both genotypes, 25 and 39 were rhythmic only in control or in cKOt mice, respectively (Figure 3B). Accordingly, the global distributions of phases (Figure 3C) and amplitudes (Figure 3D) of rhythmic metabolites were highly similar between controls and cKOt. Overall, approximately 50% of detected metabolites exhibited differential levels in control and cKOt kidneys, suggesting that the deletion of *Bmal1* has a greater effect on the abundance of metabolites than on their rhythmicity (Figure 3E). Numerous important metabolic components displayed substantial changes in their mean abundance between kidneys of control and cKOt mice, including carnitine, NAD^+^, flavin adenine dinucleotide (FAD^+^), and flavin mononucleotide (FMN) (Figure 3F). Notably, analysis of metabolite categories revealed that those related to energy production or cofactors and vitamins showed a tendency for reduced abundance, whereas lipids displayed an increased abundance in cKOt (Supplemental Table 5 and Figure 3E). In subcategories of lipids, a substantial reduction was observed for acyl-carnitines, whereas long-chain FAs and monoacylglycerols were enriched in the kidneys of cKOt mice (Supplemental Figure 2A).

Absolute quantitation and *dryR* analysis of 599 metabolites was also performed on the plasma of the same control and cKOt mice. We retained 333 metabolites detected in all samples for further analyses (Supplemental Table 6). In contrast to the kidney, a high percentage (approximately 60%) of plasma metabolites were rhythmic in control and cKO mice (Figure 3G). Most of them were identically rhythmic in the plasma of both control and cKOt mice and a few were rhythmic only in control or cKOt mice (Figure 3H). In both genotypes, rhythmic plasma metabolites displayed a unimodal phase distribution with a broad peak around Zeitgeber time 0 (ZT0) (Figure 3I). Differential analysis of plasma concentrations of metabolites from different categories revealed an increase in many phosphathidylcholines, triglycerides, and sphingomyelines in the plasma from cKOt mice (Figures 3J and Supplemental Figure 2B). However, the most important difference was observed for carnitine and its derivatives acetylcarnitine and propionylcarnitine, which were substantially reduced in cKOt, while creatinine was increased (Figure 3, K and L).

### The circadian clock in the renal tubule controls multiple pathways of NAD^+^ replenishment.

**Figure 4:**
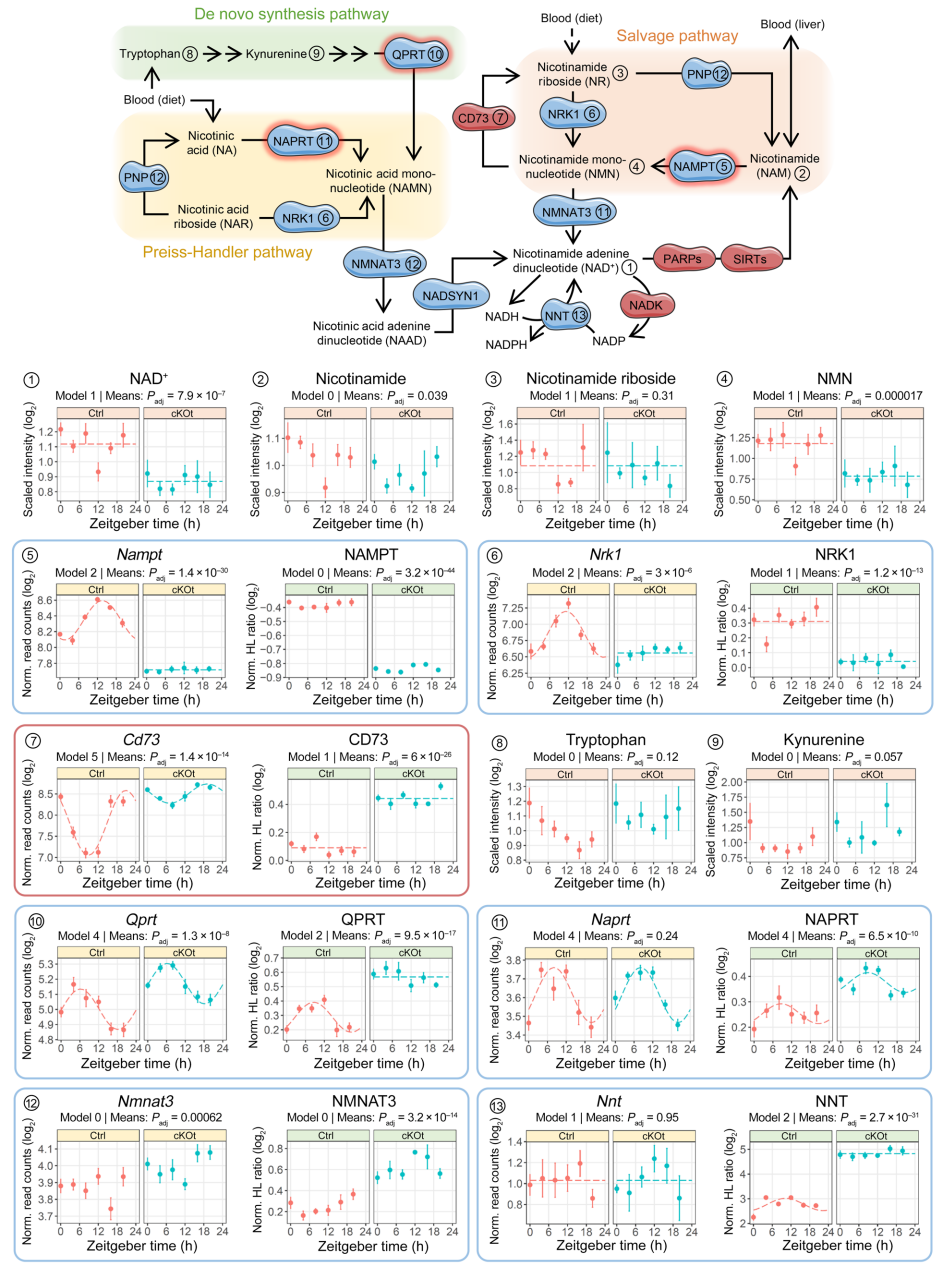
Alteration of renal NAD^+^ metabolism in cKOt mice. Schematic of the NAD^+^ metabolism depicting enzymes and metabolites involved in NAD^+^ synthesis from NAM, NAM riboside, nicotinic acid, and tryptophan (upper part). Enzymes in blue are involved in NAD^+^ synthesis, while enzymes in red are NAD^+^-consuming. Enzymes surrounded with red are rate limiting. Numbers link components of the pathway to temporal plots depicted in the lower part of the Figure. Temporal plots of major renal metabolites (peach), transcripts (yellow), and proteins (green) involved in NAD^+^ metabolism detected in renal transcriptomic, proteomic or metabolomic data sets in control (Ctrl) and cKOt mice. On each plot are mentioned the rhythmicity model and *P*_adj_ obtained, respectively, from the *dryR* comparison of rhythmicity patterns and the limma R package mean expressions comparison in Ctrl and cKOt mice. CD73, cluster of differentiation 73 also known as ecto-5′-nucleotidase; NADK, NAD^+^ kinase; NADSYN1, glutamine-dependent NAD^+^ Synthetase; NAMPT, nicotinamide phosphoribosyltransferase; NAPRT, nicotinate phosphoribosyltransferase; NMNAT3, nicotinamide nucleotide adenylyltransferase isoform 3; NNT, NADP transhydrogenase; NRK1, nicotinamide riboside kinase 1; PARPs, poly(ADP-ribose) polymerases; PNP, purine nucleoside phosphorylase; QPRT, quinolinate phosphoribosyltransferase; SIRTs, sirtuins.

Based on these observations, we focused our attention on the effect of *Bmal1* deletion on NAD^+^ metabolism. NAD^+^ is a cofactor for more than 400 enzymatic redox reactions, many of which are critical to cell metabolism. NAD^+^ biosynthetic pathways differ widely across tissues and display tissue-specific preference for different NAD^+^ precursors. Imbalance in renal NAD^+^ has been proposed as a causative factor for progression from acute kidney injury (AKI) to CKD (32, 33). Our metabolome analysis revealed a reduction in NAD^+^ levels in the kidneys of cKOt mice to a degree comparable to that observed in AKI, with a reduction of approximately 20% (34) (Figure 4). In addition, joint analysis of omics data sets showed substantial modifications in principal pathways contributing to the maintenance of intracellular NAD^+^. A dramatic reduction was observed in both mRNA and protein levels of NAM phosphoribosyltransferase (NAMPT), the rate-limiting enzyme in the NAD^+^ salvage pathway that converts NAM, a metabolite mostly derived from local NAD^+^ cleavage by NAD^+^-consuming enzymes, into NAM mononucleotide (NMN) (35). Lower expression in the kidneys of cKOt mice was also observed for the nicotinate riboside kinase (NRK1) that can produce NMN from NAM riboside (NR). Expression of CD73, an enzyme that opposes NRK1 action by converting NMN into NR was substantially increased in the kidneys of cKOt mice both at mRNA and protein levels. While the quantitative role of NRK1 in NAD^+^ production in the kidney remains unknown, this enzyme is expressed at high levels in the renal proximal tubule (36). Importantly, both *Nampt* and *Nrk1* displayed circadian clock-dependent rhythmicity, as previously shown in the liver (37). Interestingly, while the renal content of NAM is only slightly decreased and NR is not modified in the kidneys of cKOt mice, there was an approximately 30% reduction in NMN levels. The effect of impairment in salvage pathway on the NAD^+^ pool might be partially alleviated by compensatory mechanisms affecting 2 other NAD^+^ synthesis pathways, namely the de novo NAD^+^ synthesis pathway and the Preiss-Handler pathway, which, respectively, use circulating tryptophan and nicotinic acid (NA) to produce the NAD^+^ precursor NA mononucleotide (NAMN). No difference in abundance of tryptophan and kynurenine suggested that there were no alterations in the initial steps of the de novo pathway, despite downregulation of the kynurenine formamidase (AFMID) enzyme (Figure 4 and Supplemental Figure 3). However, 3 enzymes involved in the last steps of the de novo pathway, namely kynurenine 3-monooxygenase (KMO), 3-hydroxyanthranilate 3,4-dioxygenase (HAAO), and the rate limiting nicotinate-nucleotide pyrophosphorylase (QPRT) were upregulated in the kidneys of cKOt mice (Figure 4 and Supplemental Figure 3). Moreover, the rate limiting enzyme of the Preiss-Handler pathway, nicotinate phosphoribosyltransferase (NAPRT), was overexpressed, as well as NAMN adenylyltransferase 3 (NMNAT3), an enzyme converting NAMN into the NAD^+^ precursor NA adenine dinucleotide (NAAD). The purine nucleoside phosphorylase (PNP), an enzyme that mostly produces NA from NA riboside (NAR) was upregulated at both transcript and protein levels (Supplemental Figure 3). Finally, the expression of the Nicotinamide nucleotide transhydrogenase (NNT), an enzyme that produces NAD^+^ from NADH pool in mitochondria was dramatically increased at the protein level. This suggests the activation of a compensatory pathway to counteract the deficient NAD^+^ synthesis in cKOt animals.

### Impaired FA transport, carnitine shuttle, and β-oxidation in cKOt mice.

**Figure 5:**
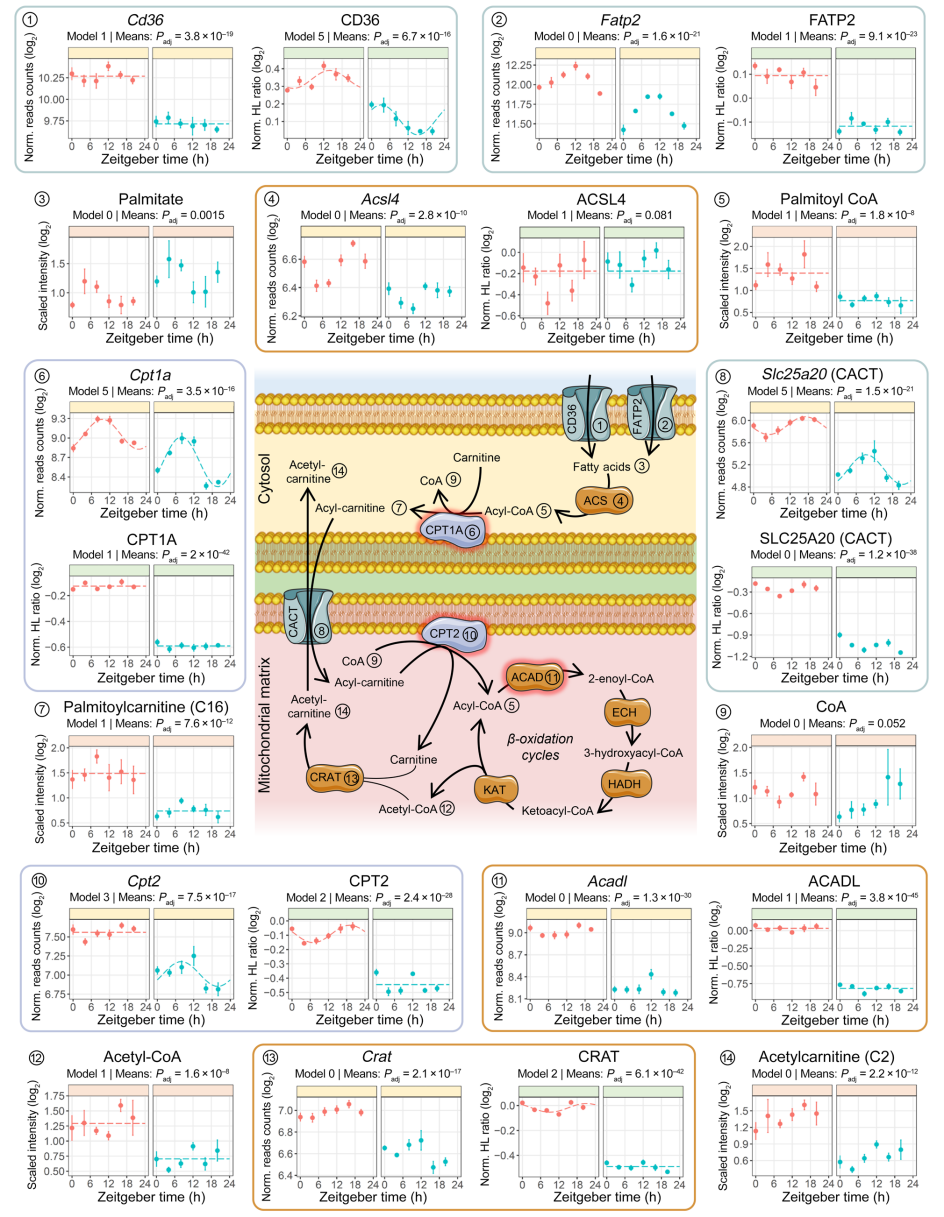
Alterations of renal fatty acid metabolism in cKOt mice. Schematic (center) and temporal plots (edges) of FA metabolism depicting major renal metabolites (peach), transcripts (yellow), and proteins (green) involved in FA entry and activation, acyl-carnitine shuttle into mitochondria, and acyl-CoA β-oxidation. Proteins surrounded with red are rate limiting. Numbers link components of the schematic to temporal plots. Rhythmicity model and *P*_adj_ obtained, respectively, from the DryR comparison of rhythmicity pattern and limma R package mean expressions comparison in Ctrl and cKOt mice are mentioned in each plot. ACADL, acyl-coenzyme A dehydrogenase, long chain; ACSL4, acyl-CoA synthetase long chain family member 4; CACT, mitochondrial carnitine/acylcarnitine carrier protein; CD36, cluster of differentiation 36 also known as fatty acid translocase (FAT); CPT1A, Carnitine palmitoyltransferase 1A; CPT2, carnitine palmitoyltransferase 2; CRAT, carnitine O-acetyltransferase; FATP2, fatty acid transport protein 2.

Both transcriptome and proteome pathway analyses and changes in the kidney tissue metabolome suggested alterations in FA metabolism in the kidneys of cKOt mice. We performed a detailed analysis of changes in the levels of mRNAs, proteins, and metabolites related to different steps of FA metabolism, the principal source of energy in the proximal tubule. As shown in Figure 5, expression of CD36 and SLC27A2 (FATP2), 2 major FA transporters in the proximal tubule (38), were reduced in the kidneys of cKOt mice at both the mRNA and protein levels. Interestingly, kidney tissue levels of FA were increased, especially during the inactive phase (Figures 5 and Supplemental Figure 4A), probably due to impairments in downstream steps of FA metabolism. Palmitoyl-CoA levels were decreased, but no clear tendency was observed for expression of different members of the acyl-CoA-synthetase family (Figure 5 and Supplemental Figure 4B). Expression of carnitine palmitoyltransferase 1A (CPT1a) and of carnitine-acylcarnitine translocase (CACT or SLC25A20), enzymes critical for the transfer of long-chain FA through the outer and inner mitochondrial membranes, respectively, were substantially reduced in the kidneys of cKOt mice (Figure 5). This reduction was paralleled by a decrease in tissue levels of majority of acylcarnitines (Figure 5 and Supplemental Figure 5A). A substantial reduction in expression was also observed for carnitine palmitoyltransferase 2 (CPT2), an enzyme that catalyzes the formation of acyl-CoAs from acylcarnitines and CoA, a step preceding β-oxidation (Figure 5). mRNA and protein expression of short-chain, medium-chain, long-chain, and very-long-chain acyl-CoA dehydrogenases (ACADS, ACADM, ACADL, and ACADVL, respectively) which catalyze the rate-limiting step of β-oxidation, were substantially decreased in cKOt mice (Figure 5 and Supplemental Figure 5B). Finally, the decrease in acetyl-carnitine levels was correlated with reduced expression of carnitine acetyltransferase (CRAT), an enzyme that catalyzes the exchange of acyl groups between carnitine and CoA. Collectively, these data demonstrated multilevel impairment in the metabolism of FA in kidneys of cKOt mice. Interestingly, reduced expression of enzymes involved in FA transport and β-oxidation was paralleled by substantial modifications in PPARs, a family of FA-activated nuclear receptors, with attenuated expression of *Pparα* and *Pparγ* and a dramatic increase in *Pparβ/δ* expression in cKOt mice (Supplemental Figure 6).

### The circadian clock in the renal tubule controls renal and systemic carnitine levels.

**Figure 6:**
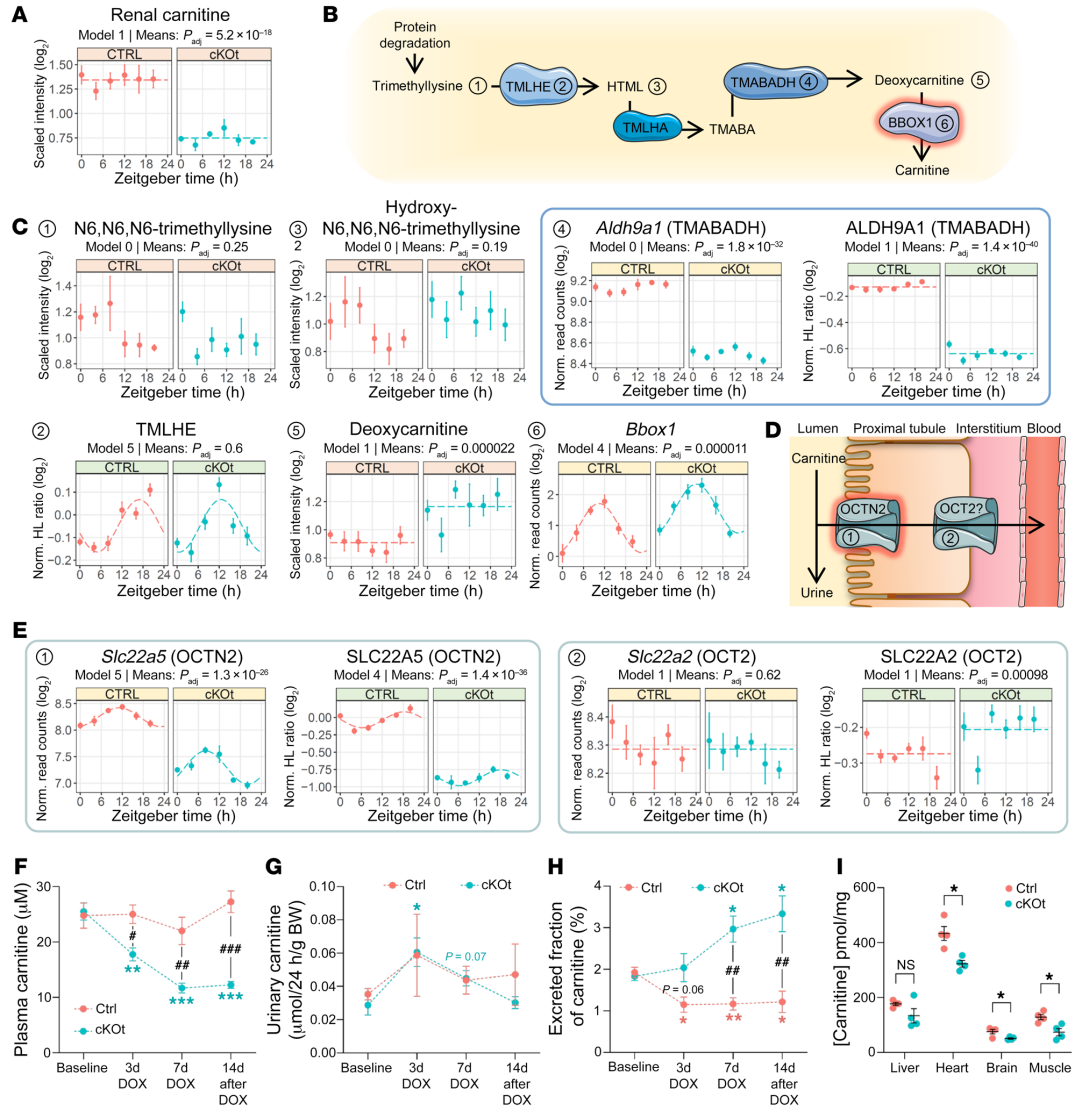
Carnitine metabolism and renal handling in cKOt mice. (**A**) Temporal plot showing the renal carnitine content in Ctrl and cKOt mice. (**B**) Schematic of renal carnitine synthesis pathway where BBOX1 (surrounded with red) is the rate limiting enzyme. (**C**) Temporal plots of major renal metabolites (peach), transcripts (yellow) and proteins (green) involved in carnitine synthesis detected in kidneys of Ctrl and cKOt mice. (**D**) Schematic of renal carnitine handling in proximal tubules: OCTN2 (SLC22A5) transporter (surrounded in red) is rate limiting for apical reabsorption of filtered carnitine. (**E**) Temporal plots showing the relative expression of renal carnitine transporters in kidneys of Ctrl and cKOt mice. Numbers link components of **B** to temporal plots. (**F**–**H**) Carnitine concentrations in plasma (**F**) or urine (**G**) and carnitine-excreted fraction (**H**) in Ctrl and cKOt mice before DOX treatment (baseline), 3 or 7 days after the beginning of the DOX treatment (3d or 7d DOX), and 2 weeks after the end of DOX treatment (14d post-DOX). (**I**) Carnitine content in liver, brain, skeletal muscle (right rectus femoris), and heart of Ctrl and cKOt mice 4 weeks after the end of DOX treatment. Throughout the Figure, the rhythmicity model obtained from *dryR* and *P*_adj_ value obtained from limma mean expressions comparison in Ctrl and cKOt mice are mentioned on temporal plots. Results in panels **F** to **I** are mean ± SEM (*n*= 4 in each genotype) determined by 2-way ANOVA and Sidak’s multiple comparison posthoc tests. OCT2, organic cation transporter 2 (OCT2); BBOX1, γ-butyrobetaine hydroxylase 1; HTML, hydroxytrimethyllysine; OCTN2, organic cation transporter novel family member 2 (SLC22A5); TMABA, trimethylaminobutyraldehyde; TMABADH, trimethylaminobutyraldehyde dehydrogenase; TMLHA, hydroxyl-trimethyl-lysine aldolase; TMLHE, trimethyl-lysine hydrolase ϵ.

The metabolome data of kidney tissue (Figure 3E and Figure 6A) and plasma (Figure 3K) revealed a substantial reduction in carnitine abundance in cKOt mice throughout the entire circadian cycle. The maintenance of intracellular carnitine levels is critical for FA oxidation and energy production. In the kidney proximal tubule, 2 mechanisms contribute to the control of intracellular carnitine levels, i.e., transcellular carnitine reabsorption and carnitine biosynthesis. These latter data confirm and extend results by Nikolaeva, et al. who showed at 2 time-points (ZT4 and ZT16) that *Bmal1* deletion results in a drop in plasma carnitine levels (12). These results tempted us to explore the role of the renal tubular circadian clock in the renal handling of carnitine and in the control of tissue carnitine levels in the kidney and other tissues. The kidney is the major site of carnitine biosynthesis (Figure 6B) (39). As shown in Figure 6C, kidney levels of N^6^-trimethyllysine (TML), the first metabolite of carnitine synthesis pathway, were not different between control and cKOt mice. Expression of N^6^-trimethyllysine dioxygenase (TMLHE) which catalyzes the transformation of TML into hydroxy-N^6^-trimethyllysine (HTML) was not different between genotypes. The identity of the second enzyme, HTML aldolase (TMLHA), which catalyzes the transformation of HTML into 4-N-trimethylaminobutyraldehyde (TMABA), remains unclear. However, expression of TMABA dehydrogenase (TMABADH or ALDH9A1), which converts TMABA into deoxycarnitine, was reduced both at the mRNA and protein levels in cKOt mice. Interestingly, deoxycarnitine levels were increased in the kidneys of cKOt mice. Finally, mRNA encoding the last and rate-limiting enzyme in the enzymatic chain, γ-butyrobetaine dioxygenase (BBOX1), was significantly increased in the kidneys of cKOt mice.

Carnitine is freely filtered by renal glomeruli and approximately 98% is reabsorbed in the proximal tubule (Figure 6D). At the apical membrane, carnitine is transported inside the cell via the sodium-coupled carnitine transporter SLC22A5 (OCTN2) (40). The basolateral carnitine extrusion is less well characterized, but a role of organic cation transporter 2 (SLC22A2 or OCT2) in this process has been proposed (41, 42). Analysis of the diurnal transcriptome and proteome revealed that both mRNA and protein expression of SLC22A5 were dramatically reduced in the kidneys of cKOt mice throughout the entire circadian cycle, whereas expression of SLC22A2 was slightly increased (Figure 6E). In a kinetic experiment, there was no difference in plasma carnitine levels between control and cKOt mice at baseline, before DOX treatment (Figure 6F). However, 3 days after the beginning of the DOX treatment, a reduction in plasma carnitine concentration was already observed in cKOt mice. Similar levels of urinary carnitine excretion in the 2 genotypes (Figure 6G) and reduction in fractional excretion of carnitine in cKOt mice (FE, Figure 6H) indicated that a reduction in plasma carnitine levels occurred in parallel with decreased carnitine reabsorption in the kidney. Analysis of tissue carnitine levels demonstrated reduction in heart, brain, and muscle, but not in the liver (Figure 6I). Despite a substantial decrease in the muscle carnitine content (approximately 50%), there was no difference in spontaneous or running-wheel activities between control and cKOt mice (Supplemental Figure 7).

### Consequences of Bmal1 deletion on mitochondrial activity.

**Figure 7:**
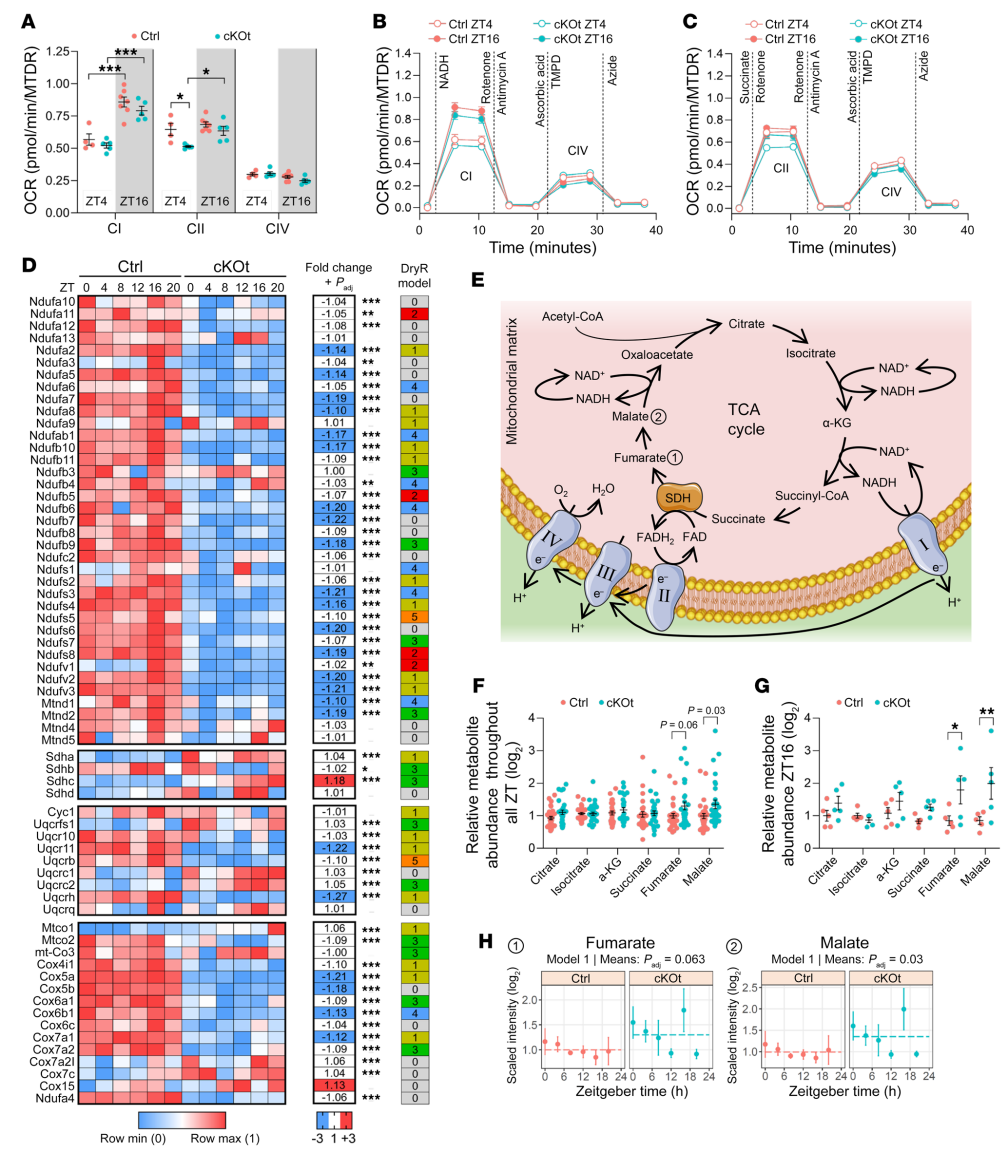
Mitochondrial activity in Ctrl and cKOt mice. (**A**–**C**) Oxygen consumption rates (OCR) of mitochondrial respiratory chain complexes I (CI), II (CII) or IV (CIV) in the kidneys of Ctrl and cKOt mice at ZT4 and ZT16. In **A**, data are presented as individual values, with mean ± SEM determined by 2-way ANOVA and Sidak’s multiple comparison posthoc tests. In **B** and **C**, data are resented as mean ± SEM and vertical dashed lines depict time-points of metabolic substrates injection during OCR measurement. (**D**) Heatmaps showing the relative expression and the fold change in mean expression of proteins forming CI to CIV complexes in the kidneys of Ctrl and cKOt mice. Statistics determined by limma comparison at 6 circadian time-points. Fold changes ≥ 1.10 are depicted in blue or red. Rhythmicity models are shown to the right of the heatmap. (**E**) Schematic of the Tricarboxylic acid (TCA) cycle and electron transport chain in mitochondrial matrix. Numbers are related to plots in **H**. (**F** and **G**) Plots showing the relative abundance of major TCA cycle metabolites at 6 circadian time points (**F**) or at ZT16 (**G**) in kidneys of Ctrl and cKOt mice. Data are individual values with mean ± SEM. In **F**, means were compared using limma and *P*_adj_ < 0.1 are shown. In **G**, means were compared using 2-tailed multiple *t* tests and resulting *P* values were corrected using the FDR approach (2-stage Step-up method of Benjamini, Krieger and Yekutieli). (**H**) Temporal plots of fumarate and malate abundance with corresponding rhythmicity model and *P*_adj_ from *dryR* and limma comparisons in kidneys of Ctrl and cKOt mice. SDH, Succinate dehydrogenase. *P < 0.05; **P < 0.001; ***P < 0.0001.

We found that cKOt mice showed deficient β-oxidation associated with deficient carnitine transport, which led to a decreased concentration of acetyl-CoA, an important fuel of the tricarboxylic acid (TCA) cycle. In addition, cKOt mice presented decreased production of NAD^+^, a key coenzyme of sirtuins including SIRT3, which is an important regulator of mitochondrial activity (43, 44). Therefore, we speculated that mitochondrial activity might be perturbed in cKOt mice. To test this hypothesis, we measured the activity of the different mitochondrial complexes at 2 time points (ZT4 and ZT16) in the kidneys of control and cKOt mice. The activity of complex IV was unaffected and the activity of complex I was rhythmic and not significantly impacted by the deletion of *Bmal1* (Figure 7, A and B). The activity of complex II was reduced in cKOt mice compared with controls at ZT4; however, it caught up to controls at ZT16, surprisingly gaining rhythmicity in cKOt mice (Figure 7, A and C). As shown in Figure 7D, the time-dependent activity of complex I was associated with rhythmicity in a number of complex I proteins (model 4), while the gain of rhythmicity of complex II was associated with a gain of rhythmicity in some proteins of the complex (model 3). The decreased activity of this complex at ZT4 could be the result of a deficient SIRT3-dependent deacetylation of the complex II catalytic subunit SDHA in cKOt mice caused by the decrease in NAD^+^ concentration (45, 46). Additional analysis of metabolomics data showed that the levels of fumarate and malate, metabolites downstream of complex II in the TCA cycle (Figure 7E), were increased in cKOt (Figure 7F). This increase was particularly significant at ZT16 (Figure 7, G and H) when SDHA was supposed to be deacetylated by SIRT3 (37). These data suggest that the TCA cycle is likely partially blocked downstream of complex II in cKOt mice. This block could also be caused by the decreased abundance of acetyl-CoA (Figure 5), which is a critical reagent for the synthesis of citrate by citrate synthase. Therefore, the decreased NAD^+^ and Acetyl-CoA synthesis in cKOt mice may both contribute to the perturbation of the TCA cycle.

## Discussion

Tissue-intrinsic circadian clocks govern a wide range of molecular processes involved in the adjustment of tissue physiology over the course of the circadian cycle (reviewed in (47)). The kidney is one of the most metabolically active organs in the body. The bulk of the energy produced by the kidney is used to fuel transepithelial reabsorption and secretion transport processes in the renal tubule. Using a multiomics approach (i.e., transcriptomics, proteomics, and metabolomics), we showed that the circadian clock in the renal tubule strongly affects several key metabolic pathways in the kidney, including NAD^+^ synthesis, β-oxidation of fatty acids, carnitine handling, and mitochondrial activity. Importantly, the spectrum of identified clock-dependent mechanisms ranges from presumably ubiquitous ones to those restricted to the kidney and a limited number of other tissues.

The characterization of these mechanisms was enabled by the unique nature of our data set. To ease access to this resource, we made all the data sets and statistical analyses available via a web application with an interactive interface (https://bix.unil.ch/circadian-kidney/). While temporal RNA-Seq data from control mice and mice deficient for circadian clock genes (total or kidney specific) have already been published (9, 48), temporal high-throughput proteomics analyses have been limited to the WT mouse liver (28, 49, 50), macrophages (30), SCN (31), and forebrain synapse (51). None of these studies were complemented with a temporal metabolomics data set. Therefore, this data set is unique, to our knowledge, and allowed a comprehensive analysis of rhythmic genes, protein expression, and associated metabolic pathways, as well as the effect of the deletion of the circadian clock regulator BMAL1 on these processes (of note, BMAL1 is a transcription factor that may have functions unrelated to the circadian clock). Compared with the liver, where only 6%–8 % of the quantified proteome is rhythmic, we found approximately 20% of rhythmic proteins in the kidney, close to what was found in the SCN or macrophages. However, a common observation in all of these studies is the critical role of posttranscriptional regulation: as in other tissues, a majority (78%) of rhythmic proteins were encoded by nonrhythmic mRNA.

The integrated omics approach allowed us to examine major pathways involved in the maintenance of intracellular NAD^+^ levels. We demonstrated that a large reduction in the NAD^+^ content in kidneys of cKOt mice was paralleled by a dramatic decrease in the expression of NAMPT, a key enzyme in NAD^+^ salvage pathway, and increased expression of QPRT and NAPRT, rate-limiting enzymes in de novo and Preiss-Handler pathways of NAD^+^ synthesis, respectively. These results suggest that decreased capacity of the salvage pathway accounts for the reduction in the NAD^+^ content in the kidneys of cKOt mice. The circadian clock regulation of NAMPT expression has been shown as one of the central mechanisms of circadian metabolic oscillations (52, 53). Our data demonstrate that this mechanism is also involved in the circadian clock–regulated adjustment of the NAD^+^ content in renal tubular cells.

FAs are the main metabolic fuel for the proximal tubule, a part of the renal tubule in which 60%–70% of tubular reabsorption and most of the tubular secretion takes place. We demonstrated that the expression of key enzymes involved in FA metabolism were substantially reduced in the kidneys of cKOt mice, particularly enzymes involved in FA uptake (CD36 and FATP2), shuttling of acylcarnitines into the mitochondria (CPT1, CPT2, CRAT, and CACT), and catalysis of the initial step of β-oxidation (ACADS, ACADM, ACADL, and ACADVL). In parallel, kidney content of palmitoyl-CoA, acetyl-CoA, and acetylcarnitine was substantially reduced in cKOt mice. For cells that cannot use glucose, this reduction suggests a substantial impairment in the energy production capacity. Regulation of different enzymes involved in FA metabolism by the circadian clock has been shown in several studies. To our knowledge, we present the first evidence that the circadian clock can influence the process of FA oxidation as a whole.

Our study provides what we believe to be novel insights regarding the role of the renal circadian clock in the control of intrarenal as well as systemic carnitine levels. Decreased intrarenal carnitine content in cKOt mice in parallel with decreased OCTN2 expression and increased fractional carnitine excretion in the urine suggested an impaired apical entry of carnitine in the proximal tubule rather than dysregulation of basolateral carnitine transport. Enhancement of the renal carnitine synthesis pathway does not compensate for this phenotype. OCTN2 is strongly expressed in the kidney, small intestine, heart, pancreas, and placenta, and, to a much lesser extent, in other tissues (54), suggesting that the effect of circadian clock–regulated OCTN2 expression may be, in part, tissue specific. A drop of approximately 50% in plasma carnitine observed in cKOt mice corresponds to the clinical feature of carnitine deficiency in people. To date, the carnitine deficiency phenotype was observed in the context of (a) primary carnitine deficiency resulting from inactivating mutations in *Octn2* gene, (b) secondary carnitine deficiency that may be caused by different metabolic abnormalities all ultimately leading to the loss of carnitine in the urine, and (c) patients with CKD undergoing hemodialysis and losing carnitine through the dialysis membrane (reviewed in ref. 55). In all 3 cases, clinical manifestations of carnitine deficiency do not occur until plasma carnitine levels drop to less than roughly 20% of normal values. Our study suggests that dysregulation of the renal tubular circadian clock could be an important aggravating factor or second hit in the progression of carnitine deficiency.

This data set allowed us, for what we believe to be the first time, to describe the role of the circadian clock on kidney mitochondrial function. Previous reports showed that the disruption of the circadian clock abrogated the mitochondrial dynamic and rhythmic activity in mouse liver, heart, skeletal muscle, and embryonic fibroblasts (56–61). Conversely, the deletion of *Bmal1* in the kidney, surprisingly, led to a gain of rhythmicity in mitochondrial complex II activity and protein expression.

There are 2 limitations to our study that should be considered. The first is that it included only males. The influence of sexual dimorphism on the circadian regulation of renal metabolism needs to be addressed in future studies. The second is related to the complexity of the cellular composition of the kidney with proximal tubule cells representing approximately 70% of kidney mass. Obviously, the circadian clock may have a different impact on metabolism in different renal cell types. For instance, our conclusions regarding the role of the circadian clock in regulation of carnitine homeostasis are limited to the proximal tubule, as OCTN2 expression is restricted to this tubular segment. In contrast, the NAMPT-dependent NAD^+^ salvage and β-oxidation pathways are ubiquitously distributed, thus leaving the possibility of a different (or no) role of the circadian clock in the regulation of these pathways in, e.g., the thick ascending limb or the distal nephron. It is well established that desynchrony between peripheral circadian clocks and environmental cycles can be induced by a variety of factors. Disease, medication, professional obligations (e.g., shift workers), or unhealthy lifestyle (e.g., irregular sleep and feeding rhythms) have been shown to strongly affect circadian rhythms in peripheral tissues. Thus, results of this study highlight the importance of rhythmic behavior for renal health. A lifestyle that aligns with diurnal changes in the environment could be even more important for patients with CKD, since this disease is per se characterized by a circadian disruption (62).

## Methods

### Animals.

All experiments were performed with male mice housed under 12-hour light/dark cycles with ad libitum access to drinking water and a standard laboratory chow diet (KLIBA NAFAG). Procedures used to generate *Bmal1*^lox/lox^/*Pax8-rtTA*/LC-1 Cre mouse strain (bred on the genetic background of the C57BL/6J mouse from The Jackson Laboratory) were described previously (12). Eight-week-old *Bmal1*^lox/lox^/*Pax8-rtTA*/LC-1 mice and their *Bmal1*^lox/lox^ littermate mice were treated for 2 weeks with DOX at 2 mg/mL in drinking water along with 20mg/mL sucrose to induce the Cre recombinase inactivation of the *Bmal1* (*Arntl*) gene. This model of tubular core-clock mechanism deletion has been previously described and validated (12). We did not predetermine sample sizes; instead, we selected group sizes based on contemporary work in the literature and accepted guidelines in the field (63). The investigators were not blinded during experiments.

For production of omics data sets, plasma and kidneys from control and cKOt mice were harvested 4 weeks after the end of DOX treatment and immediately stored at –80°C. Before freezing, both left and right kidneys were cut transversely into 2 approximately equal pieces. The 2 halves of the left kidney were used for transcriptomics and proteomics analyses and a half of the right kidney was used for metabolomics. Blood samples were collected from the tail and centrifuged to produce plasma samples. Before organ collection, mice were anesthetized with ketamine and xylasine and perfused with PBS through their abdominal aorta. A total of 72 mice were used; 6 control and 6 cKOt mice at each point, sacrificed at 6 different Zeitgeber time points: ZT0, ZT4, ZT8, ZT12, ZT16, and ZT20 (with ZT0 and ZT12 corresponding to times when light is switched on and off, respectively).

Details for carnitine measurements, physical activity experiments and respirometry experiments are described in the Supplemental Methods.

### Production of transcriptomics data set by RNA-Seq.

RNA from frozen half-kidneys of 72 mice were extracted and purified using RNAeasy MiniElute Spin Column (Qiagen). RNA quality was assessed on a Fragment Analyzer (Agilent Technologies). All RNAs had an RNA quality number (RQN) between 7.5 and 9.7. RNA-Seq libraries were prepared from 200 ng of total RNA with the Illumina TruSeq Stranded mRNA reagents (Illumina) using a unique dual indexing strategy and following the official protocol automated on a Sciclone liquid handling robot (PerkinElmer). Libraries were quantified by a fluorometric method (QubIT, Life Technologies) and their quality assessed on a Fragment Analyzer (Agilent Technologies). Clusters were generated with 2 nM of an equimolar pool from the resulting libraries using the Illumina HiSeq 3000/4000 SR Cluster Kit reagents. Sequencing was performed on the Illumina HiSeq 4000 using HiSeq 3000/4000 SBS Kit reagents for 150 cycles (single read). Sequencing data were demultiplexed, filtered for failed reads, and written to FASTQ files using the bcl2fastq2 conversion software (version 2.20, Illumina). Details for RNA-Seq reads mapping are described in Supplemental Methods.

### Data processing: normalization and RUV.

Further data processing was performed in R (version 4.0.3). Raw counts were transformed to counts per million (CPM), and genes with a low number of counts were filtered out according to the following rule: at least 1 sample in the whole data set had to have at least 1 CPM reads for a gene to be retained in the data set. Library sizes were then scaled using TMM normalization. Subsequently, the normalized counts were transformed to CPM values and a log_2_ transformation was applied using the R Bioconductor package edgeR (64).

The RUVs method from the R Bioconductor package RUVSeq was used to correct for unwanted variation in data (65). This R package offers a family of normalization methods that correct for complex unwanted technical effects of unknown origin or not aligned with the experimental design. They are based on the remove unwanted variation (RUV) strategy developed in (66, 67). The RUVs method, specifically, uses factor analysis on the differences between replicate groups of samples for estimating factors of unwanted variation, which can then be included in a linear model. As for parameter settings in the current analysis, the number of factors of unwanted variation estimated was 2 (k=2), and all genes in the normalized data set were used as control genes (default setting for control genes). A quality check by hierarchical clustering and by plotting of the 2 first principal components confirmed that sample clustering into replicate groups improved after applying the RUVs method with these parameters. The 2 genotypes are well separated; the time points of the light phase until the onset of darkness cluster together (ZT04, ZT08, and ZT12) as do the time points of the dark period until the light switches on (ZT16, ZT20, and ZT0). These quality control plots are provided for raw data, normalized data before RUVs treatment, and normalized data after RUVs treatment as Supplemental Figures 8–13.

For the subsequent statistical analysis of rhythmic patterns using the R package *dryR*, RUVs was applied to normalized logCPM data, and the corrected data table was used as input. For differential expression analysis using the R package limma (27) (see below, section “Comparison of group means”), 2 parameters estimated by RUVs were included as covariates in the linear model, while the normalized logCPM data without RUVs correction was used as expression matrix input, as is recommended by the authors of the RUVSeq package for this type of analysis.

### Sample selection for analysis.

From 6 biological replicate samples that were available per time point and genotype, 5 were selected for further data analysis, which allowed for the removal of outliers and reduced the size of the RNA-Seq data set from 72 to 60 samples (6 time points × 2 genotypes × 5 replicates). Selection criteria were the RNA integrity number (RIN) from the Agilent Bioanalyzer system, which measures RNA quality and distance from the other replicate samples in principal component analysis (PCA) or multi-dimensional scaling (MDS) plots. Supplemental Table 8 displays decision criteria and RIN values for all 72 mice. The same 60 individual mice chosen were then also used to procure samples for proteomics and metabolomics analyses in order to have matching data sets.

### Production of proteomics data set by LC-MS.

The kidney proteomics data set has been produced using 60 half-kidney samples from control and cKOt mice sacrificed at 6 different circadian times. The 60 samples were processed in 5 batches of 12 (each time point had 6 controls and 6 cKOt mice), with each batch corresponding to a full time series for each genotype. Details for tissue extraction, trypsin digestion, sample cleanup, and fractionation and liquid chromatography–mass spectroscopy (LC-MS) analysis are described in Supplemental Methods.

### Protein identification and quantification.

Raw MS data were processed by the MaxQuant software (version 1.6.14.0) integrating the Andromeda search engine (68). The SWISSPROT mouse proteome database of September 19^th^, 2020, including validated splice variants was used (25,321 sequences), with sequences of common contaminants added. FDR filtering of both peptide spectrum matches (PSM) and protein identifications was fixed at 1%. Search parameters allowed for 2 missed cleavages and protease specificity was set to trypsin (K, R) with cleavage after prolines included. Carbamidomethyl on cysteines was set as fixed modification, and acetyl at the protein N-terminal and oxidation on methionines as variable modification. The SILAC-labeled kidneys were frozen at –80°C until time of extraction. Quantitation of individual protein intensities relative to the reference was performed by MaxQuant as described (68) and was based on the median ratio of peptides for each protein. Global normalization of total protein intensities relative to the heavy standard in each heavy:light mix (to correct for uneven mixing ratios) was also performed automatically by the software as part of the standard MaxQuant workflow for SILAC quantitation (68). Initial mass precursor tolerance was 20 ppm and was then dynamically adjusted to 5–6 ppm by MaxQuant after recalibration, and fragment mass tolerance was fixed at 0.5 Da. The MaxQuant output file proteinGroups.txt was further processed with the Perseus software (69). We used SILAC ratios normalized internally by MaxQuant for all further analyses. Proteins only identified by modified peptides, reverse hits, and known contaminants were eliminated, and all SILAC ratios were log_2_ transformed. The resulting raw table contained 6,993 protein groups.

### Data processing: imputation and RUV.

Further data processing was performed in R (version 4.1.0). The proteomics data set had 34% missing data values. Proteins with fewer than 48 out of 60 data values (3,164 features) were removed from the data set prior to statistical analysis. Also, proteins that did not have at least 2 peptides used in quantification (data column “razor+unique” < 2) were removed (272 features). The resulting filtered data table contained 3,809 features. The missing values in this filtered data table were imputed with the R package missForest with default settings (70), using a random forest trained on the available data values to predict the missing data points.

An RUV normalization was applied to the proteomics data to correct for batch effect and unwanted variation of unknown sources. All RUV correction steps described hereafter were performed with the method RUVIII from the R package ruv, which relies on replicate groups like the method RUVs that were used for RNA-Seq data. We used all features as negative control features.

We employed a hierarchical approach similar to the inter-batch correction strategy presented in (71), which was implemented in the R package hRUV and was developed for large omics data sets with batch effect. This publication introduced the concept of sequential batch correction: instead of treating a large data set all at once for batch correction, one can start with a subset of the data and correct only this, then add more and more batches sequentially for several correction rounds, allowing one to dynamically change normalization factors from round to round. The authors propose 2 tree-structured approaches (balanced and concatenating) for sequential, hierarchical merging of batches. We used a mix of the 2 types of structures in a 2-level strategy. In a first step, the data was divided into cKOt and control sample subsets and a concatenation strategy was applied to the samples from each genotype separately. There were 5 batches of 6 samples from each genotype. We started with 3 batches to create a starting data set large enough for an RUV normalization and applied RUVIII to it, then added the fourth batch and ran RUVIII again, then included the fifth batch and ran RUVIII once more. The number of factors of variation to estimate was set to k=3, k=4, and k=5, respectively, in the 3 rounds of RUVIII (number of batches that were included in each round). In a second step, we combined the corrected data from the 2 genotypes and performed RUVIII for a final correction, with k=2. We conceived this step as a data merging procedure with a simple balanced structure. As a quality control and aid in fine-tuning the details of our approach, hierarchical clustering, plots of the first 2 principal components, and RLE plots were generated and used to visually assess improvement in sample clustering and reduction in variability. After completion of this 2-level RUVIII–based normalization, the initially imputed values were removed, then reimputed using the now-normalized data. Plots of hierarchical clustering and of principal components before RUV treatment and after RUV treatment with reimputation are provided in Supplemental Figures 14–17.

### Mapping of proteins to genes.

Details are described in Supplemental Methods.

### Production of renal metabolomics data set.

Kidney and plasma metabolomics data sets have been produced using 60 half-kidney samples or 60 plasma samples, from control and cKOt mice sacrificed at 6 different circadian times. Kidney metabolomics data were produced by Metabolon, according to its standard methods (see details in Supplemental Methods). Before statistical analysis, metabolites with more than 20% missing data values across all samples were removed from the data set. The remaining missing values were imputed with the R package missForest with default parameters. Data was treated with glog2 from the R package MKmisc for variance stabilization, and, as a final processing step, data imputation with missForest was recomputed with the transformed data. The data table used for statistical analysis contained 814 metabolites.

### Production of plasma metabolomics data set.

Plasma metabolomics data were produced by Biocrates according to its standard operating procedures and state-of-the-art techniques, using the MxP Quant 500 kit (see details in Supplemental Methods). Metabolites with missing data values were removed from the data set, making data imputation unnecessary. The data table used for statistical analyses contained 332 metabolites plus a metabolite ratio (kynurenine/tryptophan).

### Respirometry.

Details for respirometry experiments are described in Supplemental Methods.

### KEGG pathway over-representation analysis.

Details are described in Supplemental Methods

### Data availability.

All RNA-Seq raw data sets generated in this work have been deposited into the Gene Expression Omnibus (GEO) database (GSE216252). The mass spectrometry proteomics data have been deposited to the ProteomeXchange Consortium via the PRIDE partner repository (proteomexchange.org) with the data set identifier PXD036803 (https://proteomecentral.proteomexchange.org/cgi/GetDataset?ID=PXD036803). The mass spectrometry metabolomics data (2 data sets from kidney and plasma, respectively) have been deposited to the Zenodo repository (https://zenodo.org/record/7225427#.ZBRTVhWZPap). All reagents, softwares, mouse lines, and data repository information are available in Supplemental Table 7.

### Statistics.

For differential rhythmicity analysis, analysis of rhythmic patterns was performed using the R package *dryR* (27). *dryR* performs differential rhythmicity analysis of omics data sets with 2 or more sample groups. The present study used a 2-group design (i.e., 2 genotypes: control and cKO); in this scenario, *dryR* fits 5 models to each feature and selects a model using the Bayesian information criterion (BIC). Model 1 had no rhythmicity in either group; model 2 had rhythmic genes in control mice and nonrhythmic genes in cKO (loss of rhythm); model 3 had nonrhythmic genes in control, rhythmic genes in cKO (gain of rhythm); model 4 had rhythmic genes in both groups with identical acrophase and amplitude (unaltered rhythm); model 5 had rhythmic genes in both groups with differing acrophase or amplitude between groups (altered rhythm).

For all data sets including RNA-Seq, proteomics, metabolomics in kidney, and metabolomics in plasma, preprocessed normalized data were analyzed using *dryR*’s drylm function. This function expects normally distributed data and internally uses the base R function lm for fitting the sinusoidal curves.

Internal to drylm, a model selection method, based on the BIC, is employed to determine the best-fitting model for each feature. Starting from the BICs from all 5 models, Schwartz weights (BICW) are calculated for the models, and the one with the greatest BICW is retained. The BICWs give an indication of how well a model’s BIC is distinguished from the lowest BIC among the 5 models. The 5 BICWs are used as a measure of confidence in the model that was selected for a particular feature. A threshold can be applied to the BICWs to separate out features with low-confidence model assignment. In the present study, features with BICW of less than 0.65 for the best-fitting model were considered not classifiable (“ambiguous” model). Details for comparison of group means are described in Supplemental Methods. Comparisons of more than 2 means were performed by 2-way ANOVA and Šidák’s multiple comparison posthoc tests or by using 2-tailed multiple *t* tests and the FDR approach of *P* value correction.

### Study approval.

Experiments with animals were performed in accordance with the Swiss guidelines for animal care, which conform to the NIH animal care guidelines and approved by Swiss cantonal (Canton de Vaud) and federal veterinary authorities (authorization #3488 to DF).

## Author contributions

YB, FG, FA, and DF conceptualized the project. YB, CA, GC, FD, and SL performed the experiments. LW, LG, MQ, SP, MI, BDW, and MW curated the data and performed the formal analysis. FG and DF acquired funding for the project. DF wrote the original draft of the manuscript. YB, LW, FG, BDW, MW, SL, FA, and DF reviewed and edited the manuscript. The order of the co–first authors was decided based on scientific contribution to the paper. All authors reviewed and approved the final version of the manuscript.

## Supplementary Material

Supplemental data

Supplemental table 1

Supplemental table 2

Supplemental table 3

Supplemental table 4

Supplemental table 5

Supplemental table 6

Supplemental table 7

Supplemental table 8

## Version 1 03/02/2023

In-Press Preview

## Version 2 04/17/2023

Electronic publication
