# Supplementary material for: Multiomics reveals multilevel control of renal and systemic metabolism by the renal tubular circadian clock
Source: J Clin Invest. 2023 Apr 17;133(8):e167133. doi: 10.1172/JCI167133 (PMC10104904; doi:10.1172/JCI167133)
Supplement: Supplemental data [file jci-133-167133-s075.pdf]

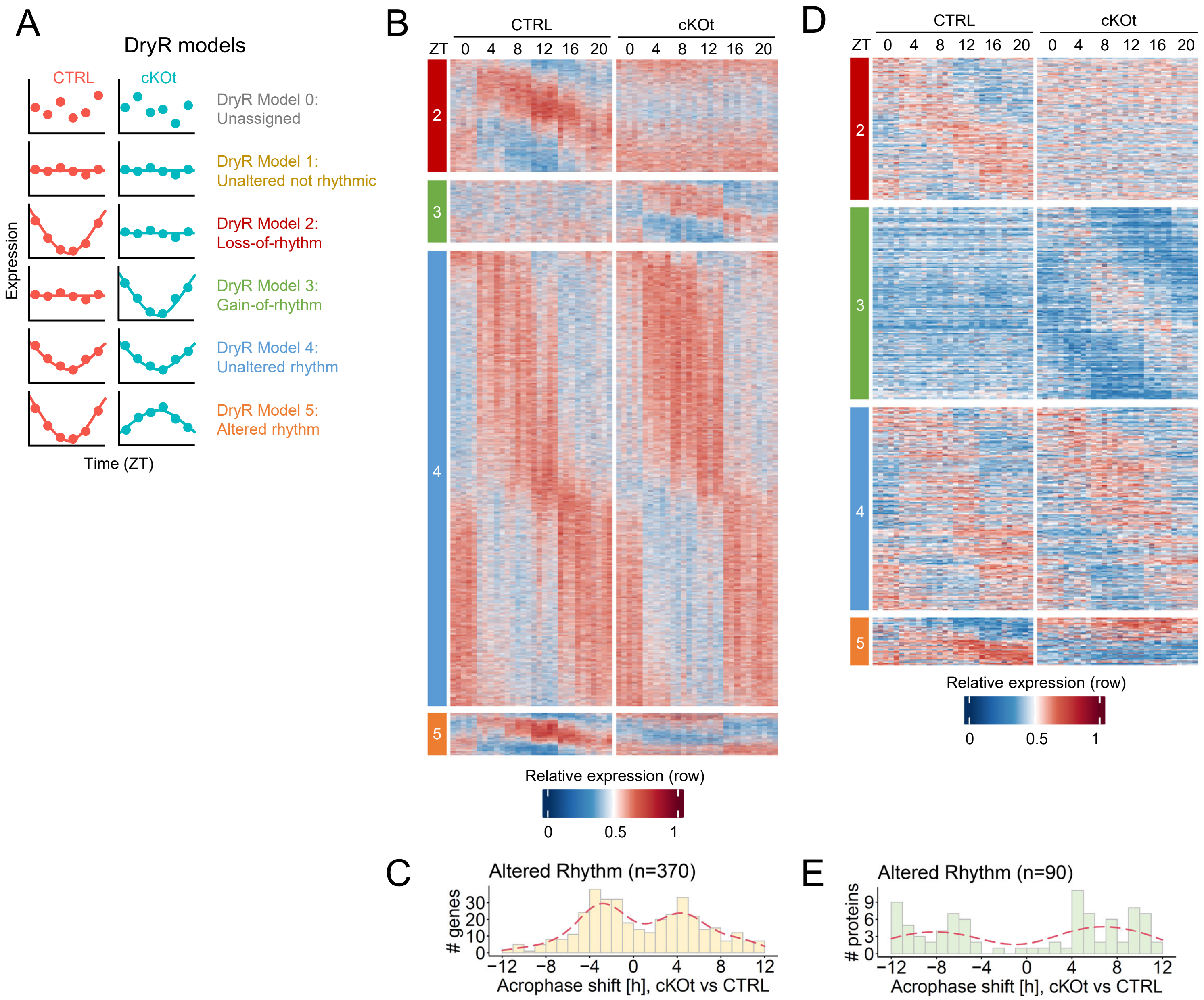

Figure S1: DryR rhythmicity models and acrophase changes in renal transcriptome and metabolome of cKOt mice.
A. Circadian rhythmicity patterns of transcripts, proteins or metabolites in CTRL and cKOt mice that correspond to each DryR rhythmicity model. DryR determines the best fit between line and sinusoidal curve for temporal expression of each component of each omic dataset throughout the circadian cycle. Then DryR compares fits from CTRL and cKOt mice to assign component to one of its 6 rhythmicity models. Components whose circadian rhythmicity patterns are unclear in CTRL and/or cKOt mice are “Unassigned”. In DryR model 1, circadian patterns of expression fit best with a line in both genotypes. In DryR model 2, circadian patterns of expression fit best with a sinusoidal curve in CTRL mice and with a line in cKOt mice. In DryR model 3, circadian patterns of expression fit best with a line in CTRL mice and with a sinusoidal curve in cKOt mice. In DryR model 4, circadian patterns of expression fit best with a sinusoidal curve in both genotypes. In DryR model 5, circadian patterns of expression fit best with a sinusoidal curve in both genotypes but the acrophase and/or amplitude of the fit is different between CTRL and cKOt mice. **B.** Heatmap showing the relative expression of transcripts assigned to DryR rhythmicity models 2, 3, 4 and 5 along the circadian cycle in CTRL and cKOt mice. **C.** Histogram showing the distribution of the acrophase shift between CTRL and cKOt mice for transcripts assigned to DryR rhythmicity model 5. Red dashed lines: kernel density estimates. **D.** Heatmap showing the relative expression of proteins assigned to DryR rhythmicity models 2, 3, 4 and 5 along the circadian cycle in CTRL and cKOt mice. **E.** Histogram showing the distribution of the acrophase shift between CTRL and cKOt mice for proteins assigned to DryR rhythmicity model 5. Red dashed lines: kernel density estimates. cKOt refers to Bmal1$^{lox/lox}$/Pax8-rtTA/LC1 mice and CTRL refers to Bmal1$^{lox/lox}$ Control mice.

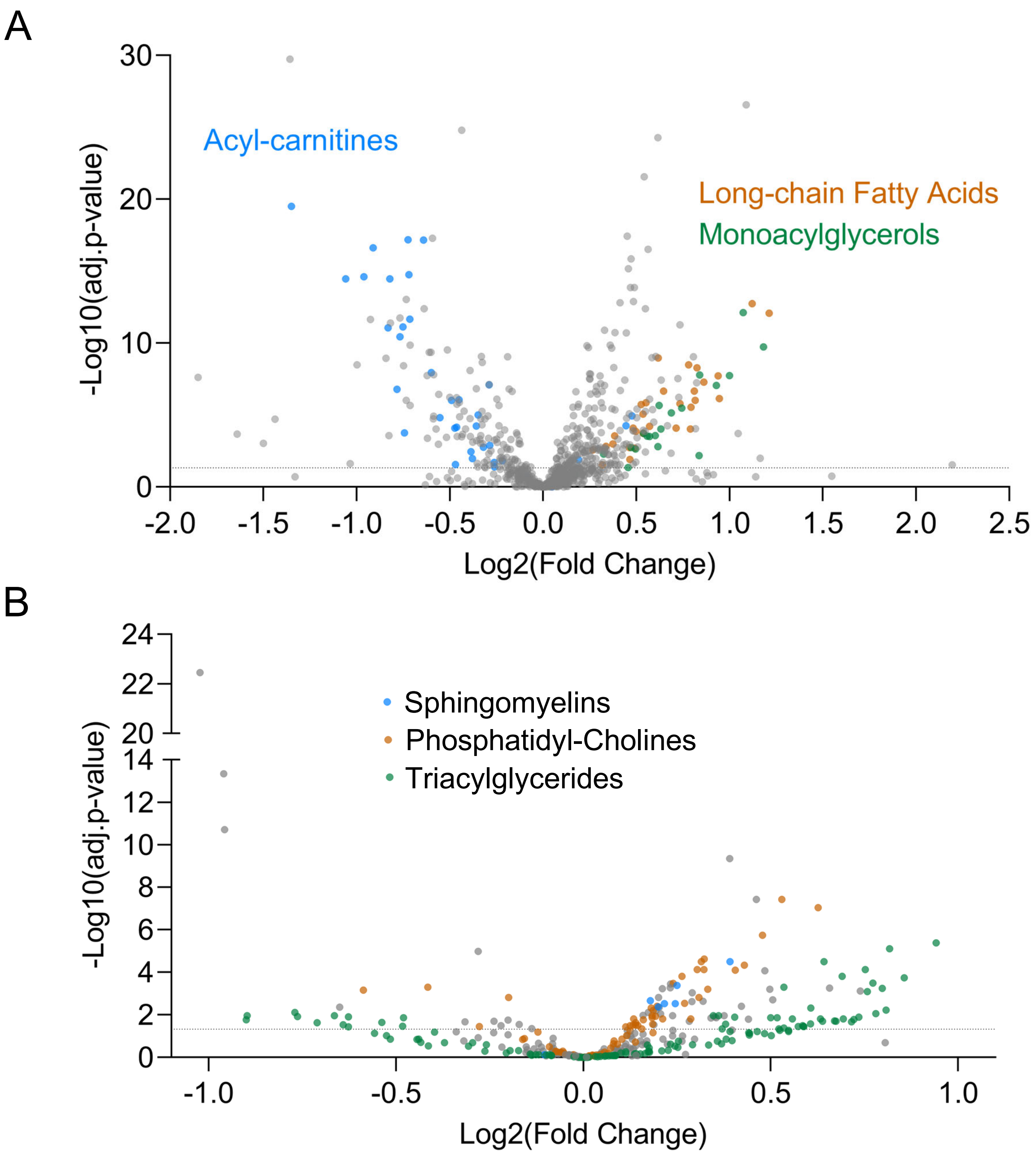

Figure S2: Main classes of renal and plasma metabolites altered in their mean expression level in cKOt mice.
A. Volcano plot of renal metabolites spread by their Log2 Folc-change of mean expression throughout the circadian cycle in cKOt mice as compared to control mice, in function of their -Log10 adjusted p-value obtained from Limma comparison of means. Acyl-carnitines, Long-chain fatty acids and Monoacylglycerols correspond to blue, orange and green dots respectively. The horizontal dotted line corresponds to the limit above which –Log10 adjusted p-values are considered significants (equivalent of adjusted p-value < 0.05 before data transformation). **B.** Volcano plot of plasma metabolites spread by their Log2 Fold-change of mean expression throughout the circadian cycle in cKOt mice as compared to control mice, in function of their -Log10 adjusted p-value obtained from Limma comparison of means. Sphingomyelins, Phosphatidyl-cholines and Triacylglycerides correspond to blue, orange and green dots respectively. The horizontal dotted line corresponds to the limit above which –Log10 adjusted p-values are considered significants (equivalent of adjusted p-value < 0.05 before data transformation).

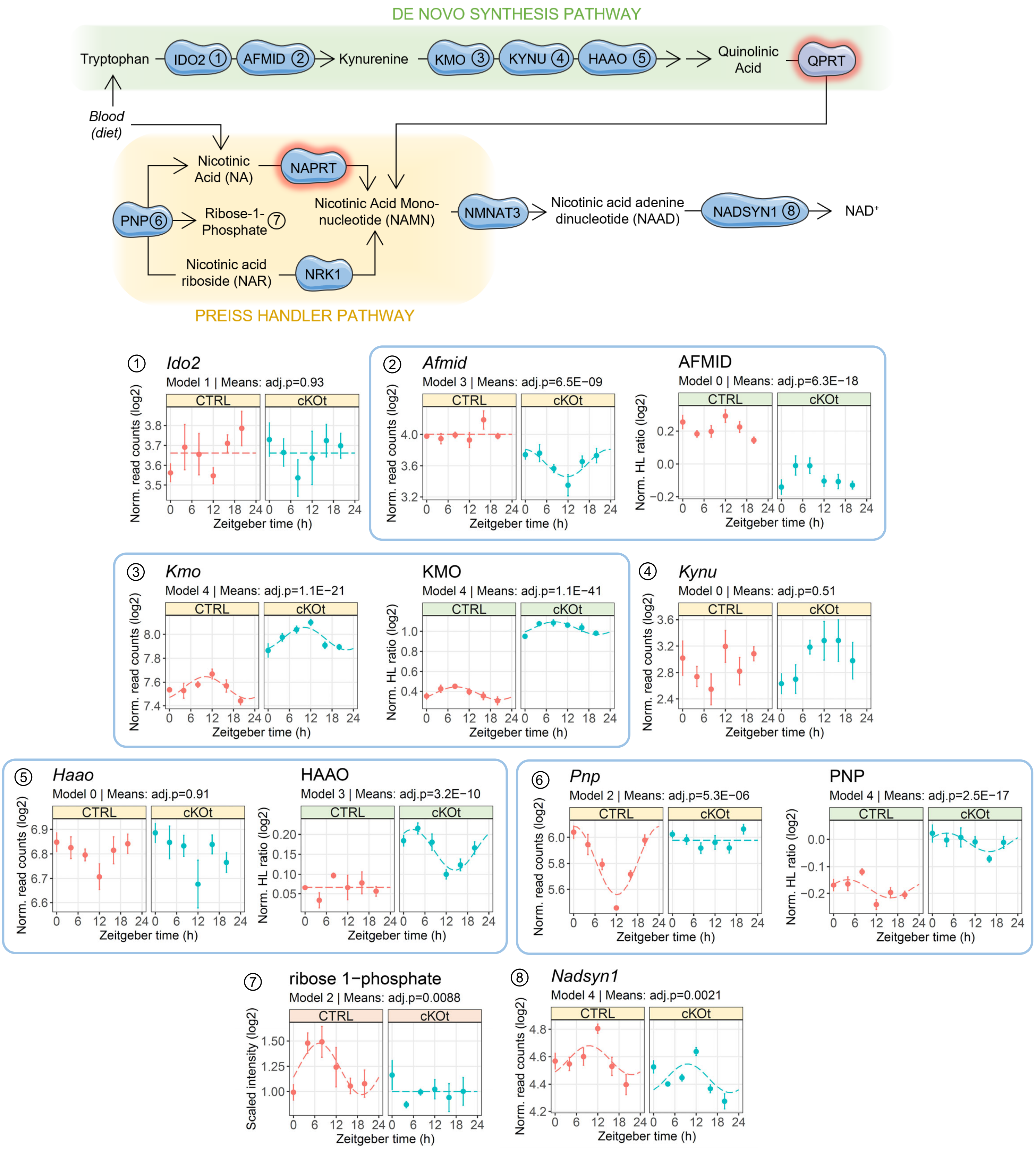

Figure S3: Renal de Novo NAD+ synthesis pathway and Preiss-Handler pathway in cKOt mice. A. Detailed schematic of the de novo NAD+ synthesis and Preiss Handler Pathway (upper panel). Enzymes glowing in red are rate limiting. Numbers link components of the pathway to temporal plots (lower panels). Temporal plots depict additional renal metabolites ( ), transcripts ( ) and proteins ( ) involved in NAD+ metabolism that have been detected in renal transcriptomic, proteomic or metabolomic datasets in CTRL and cKOt mice. Rhythmicity model and adjusted p-value obtained respectively from the DryR comparison of rhythmicity patterns and Limma mean expressions comparison in CTRL and cKOt mice are mentioned on each plot. cKOt refers to Bmal1$^{lox/lox}$/Pax8-rtTA/LC1 mice and CTRL refers to Bmal1$^{lox/lox}$ Control mice. AFMID: Kynurenine formamidase ; HAAO: 3-Hydroxyanthranilate 3,4-Dioxygenase ; IDO2: Indoleamine 2,3-dioxygenase 2 ; KMO: Kynurenine 3-Monooxygenase ; NADSYN1: Glutamine-dependent NAD(+) synthetase ; PNP: Purine nucleoside phosphorylase.

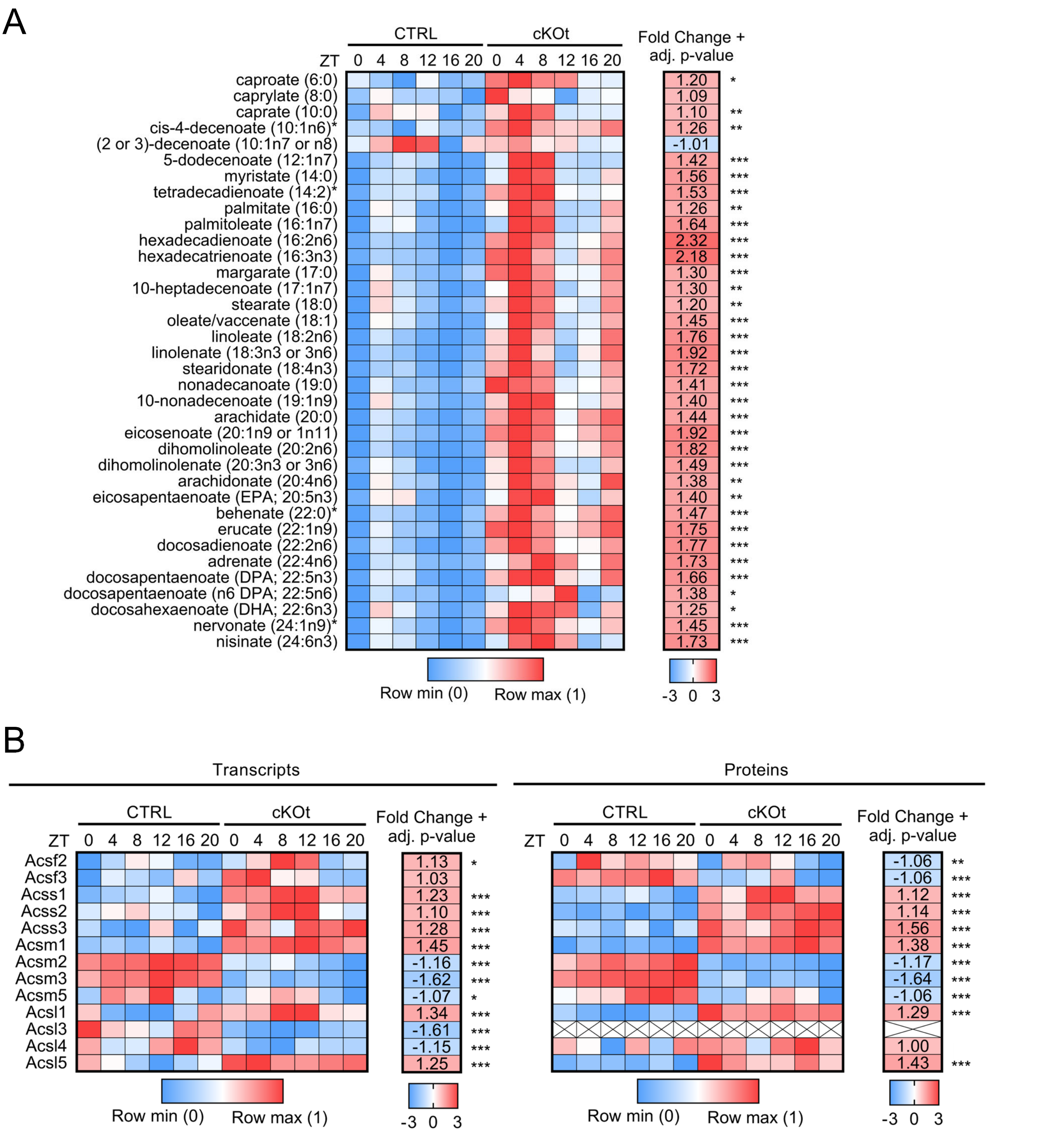

Figure S4: Expression of Fatty-acids and Acyl-CoA Synthetases in CTRL and cKOt mice. **A.** Heatmap showing the relative abundance and the fold-change in mean abundance throughout the circadian cycle for all detected fatty acids in renal metabolomes of CTRL and cKOt mice. Metabolites are identified by their biochemical name and sorted by the length of their carbon chain backbone. **B.** Heatmaps showing the relative expression and the fold-change in mean expression for all detected Acyl-CoA Synthetase at transcriptional (left panel) and protein (right panel) levels in CTRL and cKOt mice. Enzymes are identified by their gene name and sorted by Alphabetical order. ***, ** and * symbols indicate that adjusted p-value obtained by the Limma comparison of mean expressions in CTRL and cKOt throughout the circadian cycle is <0.05, >0.01 or >0.001 respectively. Missing data from Acsl3 protein expression have been replaced by crossed white cells. cKOt refers to Bmal1$^{lox/lox}$/Pax8-rtTA/LC1 mice and CTRL refers to Bmal1$^{lox/lox}$ Control mice.

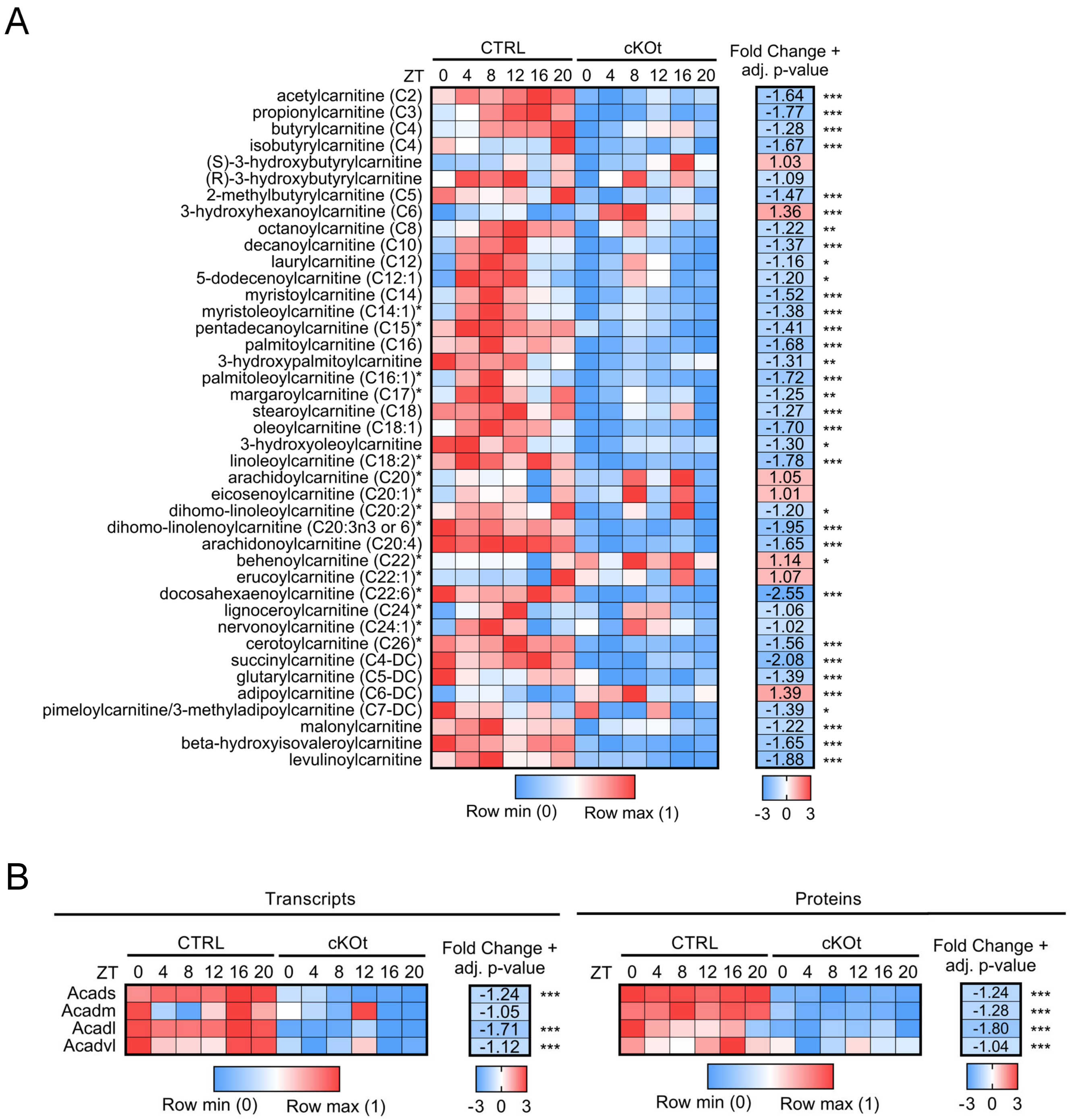

Figure S5: Expression of acylcarnitines and Acyl-CoA dehydrogenases in CTRL and cKOt mice. **A.** Heatmap showing the relative abundance throughout the circadian cycle and the fold-change in mean abundance for all detected acylcarnitines in renal metabolomes from CTRL and cKOt mice. Acylcarnitines are identified by their biochemical name and sorted by the length of their carbon chain backbone and their classes. **B.** Heatmaps showing the relative expression and the fold-change in mean expression for major Acyl-CoA dehydrogenases at transcriptional (left panel) and protein (right panel) levels in CTRL and cKOt mice. Enzymes are identified by their gene name and sorted by the length of their substrate's carbon chain backbone. ***, ** and * symbols indicate that adjusted p-value obtained by the Limma comparison of mean expressions in CTRL and cKOt throughout the circadian cycle is <0.05, >0.01 or >0.001 respectively. cKOt refers to Bmal1$^{lox/lox}$/Pax8-rtTA/LC1 mice and CTRL refers to Bmal1$^{lox/lox}$ Control mice.

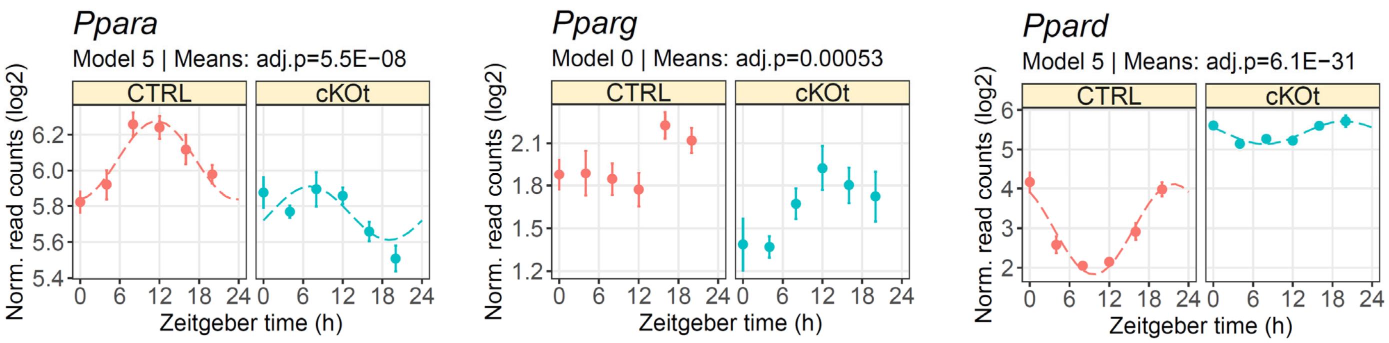

Figure S6: Alteration of Peroxisome Proliferator-Activated Receptors in kidneys of cKOt mice. Temporal plots showing the pattern of transcriptional expression throughout the circadian cycle, the DryR rhythmicity model and the adjusted p-value obtained by Limma mean expressions comparison for genes encoding for alpha (left), gamma (middle) and delta (right) Peroxisome Proliferator-Activated Receptor (PPAR) in kidneys of CTRL and cKOt mice.

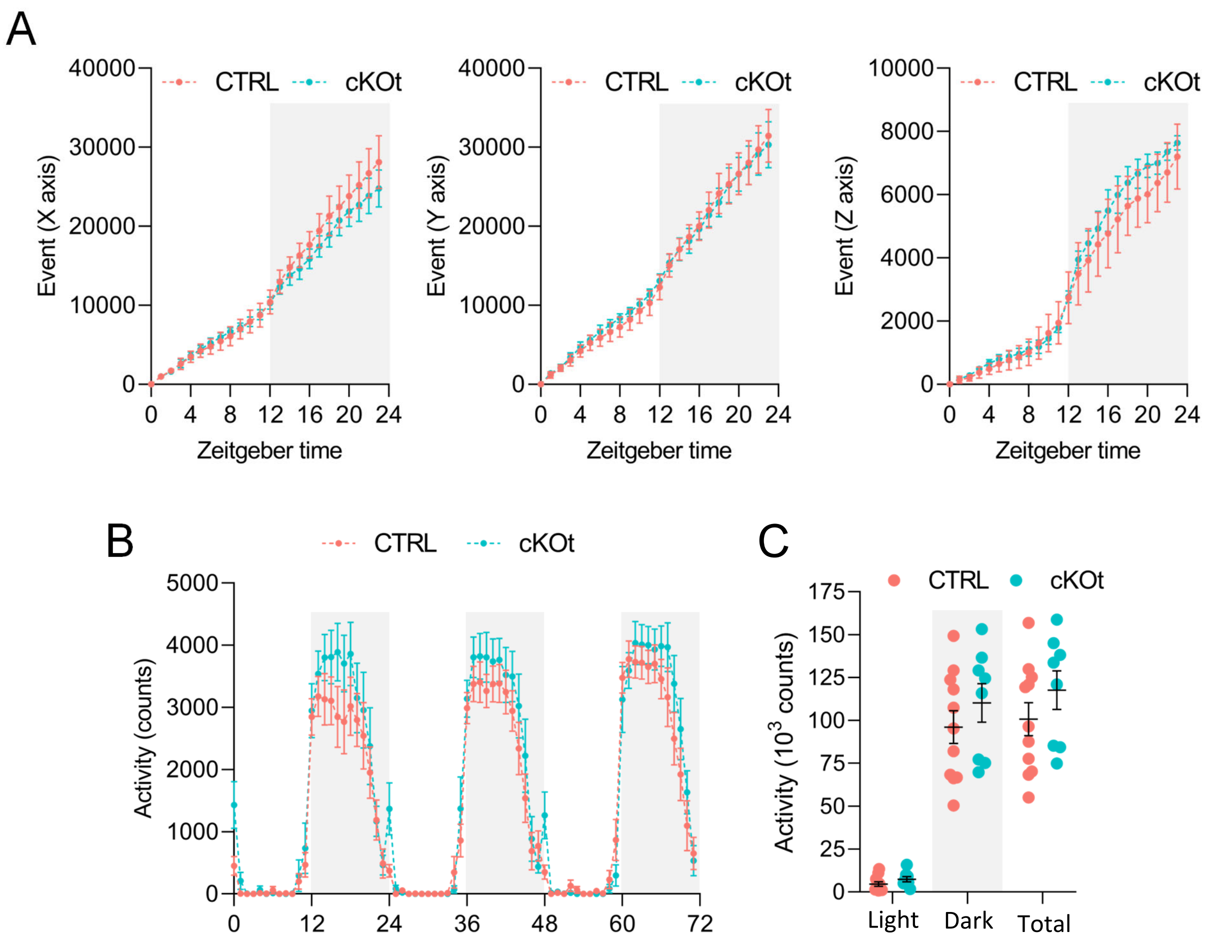

Figure S7: Measurements of the physical activity throughout the circadian cycle in CTRL and cKOt mice.
A. Cumulative plots of CTRL and cKOt mice movements recorded in 3 dimensions (X, Y and Z axis) during the circadian cycle. The dark phase of the circadian cycle (ZT12 to ZT0) is depicted with a light grey background. Results from 4 CTRL mice and 4 cKOt mice are presented as means connected by dotted lines +/- standard error of the mean. **B.** Plot showing variations in the physical activity of CTRL and cKOt mice housed in cages containing running wheels over three days under light-dark cycles. The number of wheel revolutions per hour (counts) is used as a proxy of the physical activity. The dark phase of the circadian cycle (ZT12 to ZT0) is depicted with a light grey background. Results from 11 CTRL mice and 8 cKOt mice are presented as means connected by dotted lines +/- standard error of the mean. **C.** Plot showing the mean physical activity (counts) of CTRL and cKOt mice housed in cages containing running wheels during the day (light phase), night (dark phase) or during the whole circadian cycle. Results from 11 CTRL mice and 8 cKOt mice are presented as individual values. Mean +/- standard error of the mean are plotted as black line and error bars respectively.

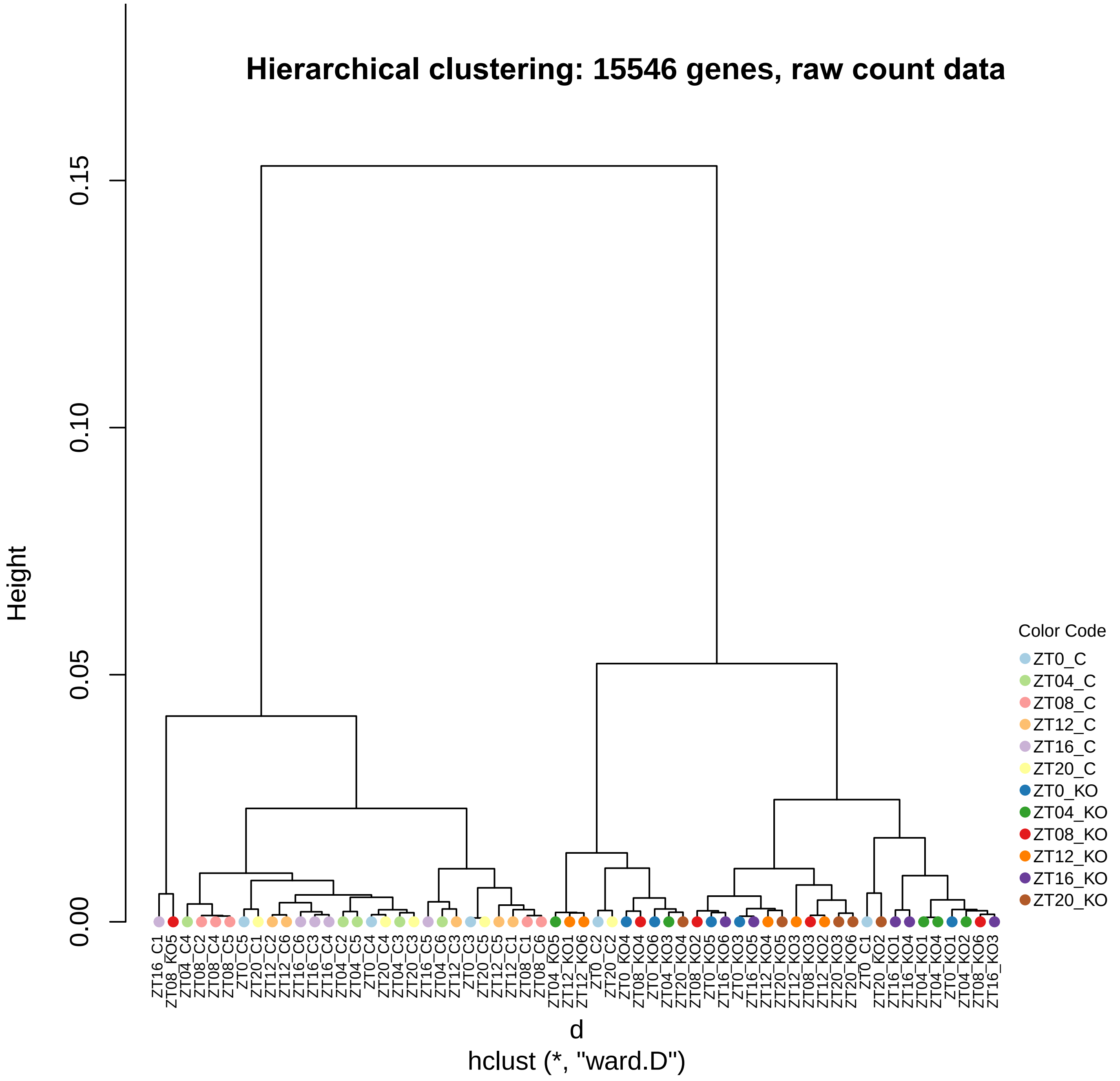

Figure S8: Hierarchical clustering of RNA-Seq raw count data. Genes were filtered for low expression (at least 1 CPM reads in at least one sample). In raw data, samples do not cluster neatly by experimental groups.

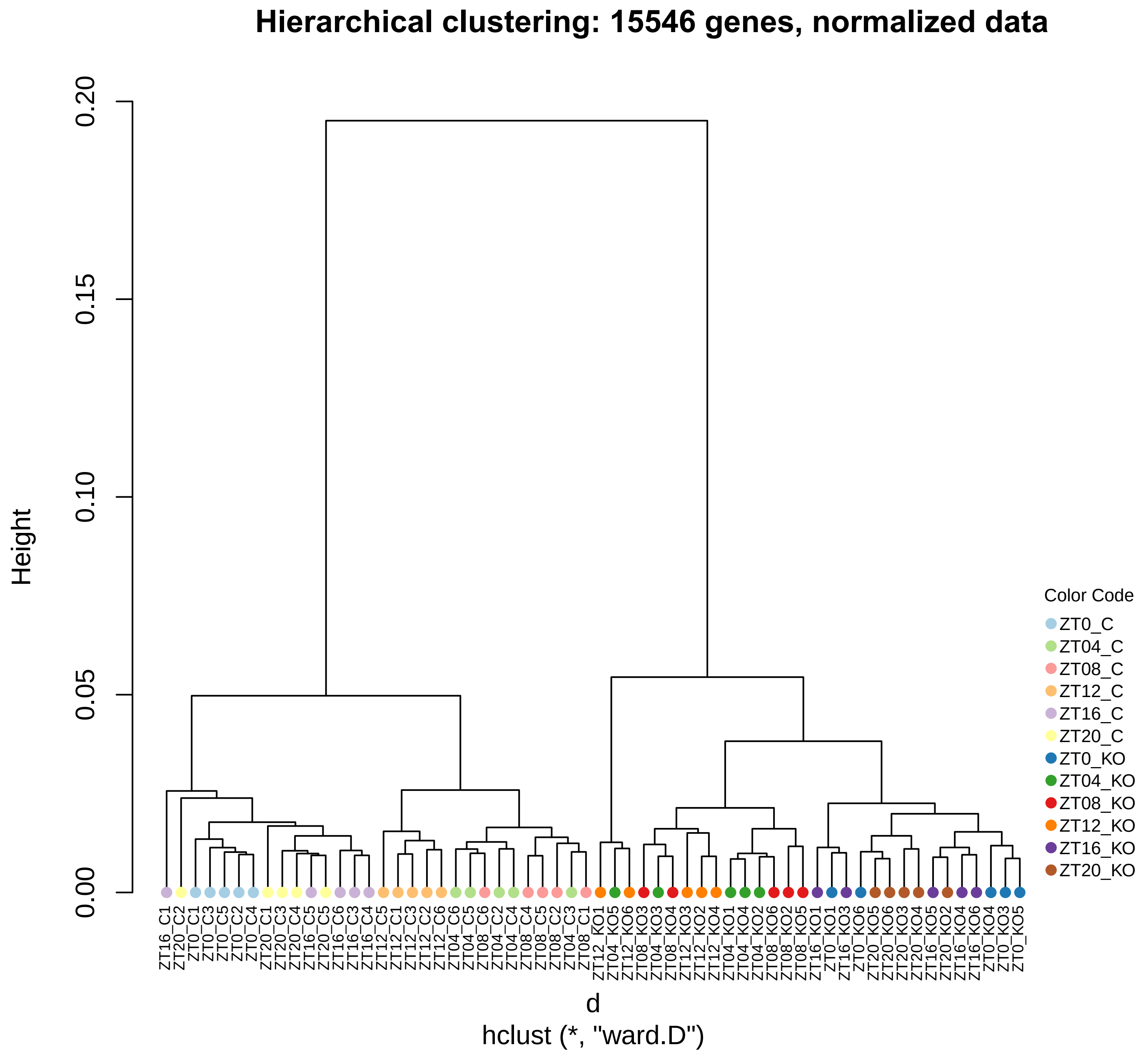

Figure S9: Hierarchical clustering of RNA-Seq normalized data (scaled using the TMM method and transformed to logCPM). Genotypes (C = CTRL, KO = cKOt) are well separated.

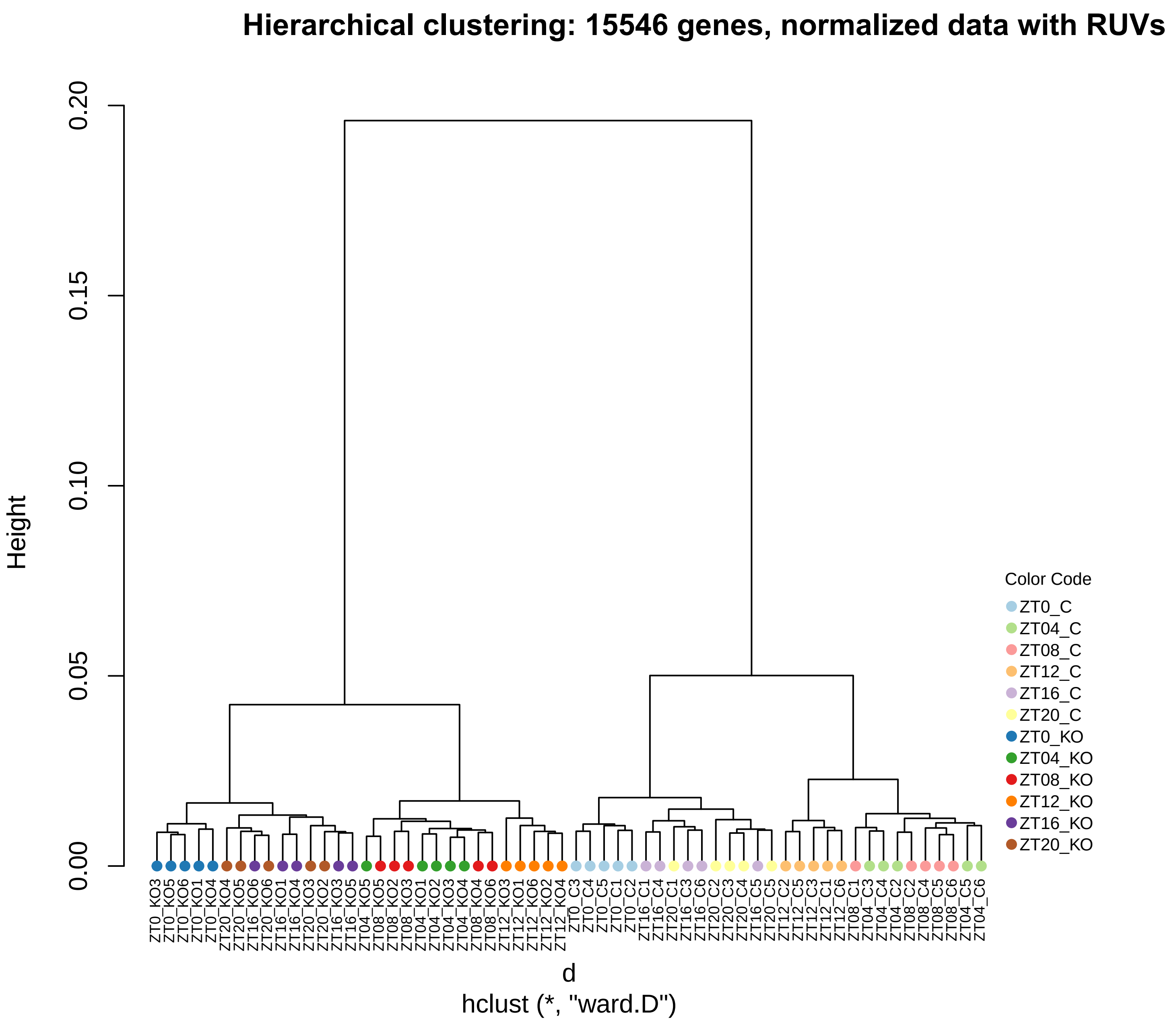

Figure S10: Hierarchical clustering of RNA-Seq normalized data with RUVs to remove unwanted variation (k=2). Genotypes (C = CTRL, KO = cKOt) are well separated. Time points (ZT) cluster by light and dark phase.

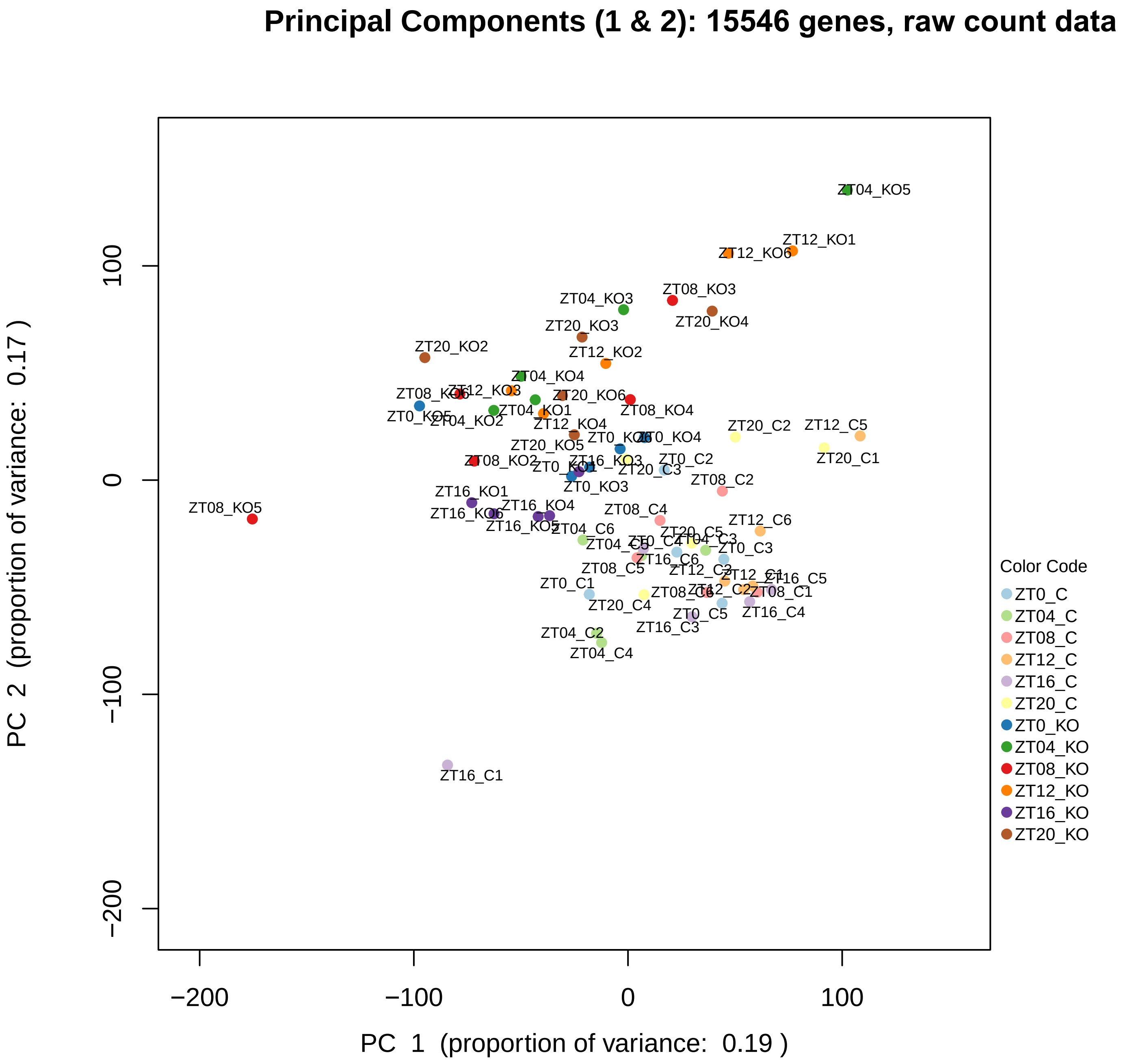

Figure S11: First two principal components of RNA-Seq raw count data.

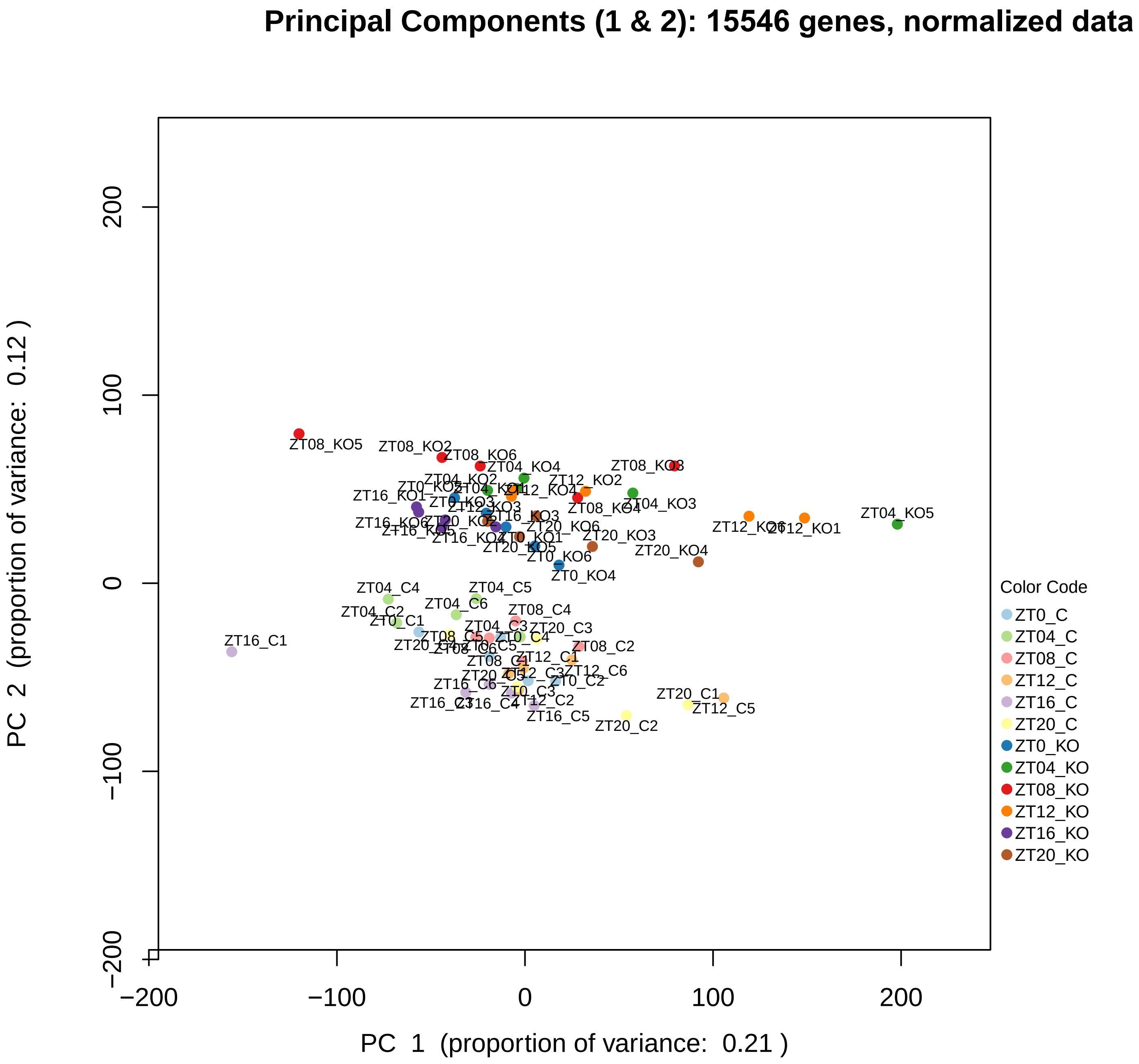

Figure S12: First two principal components of RNA-Seq normalized data.

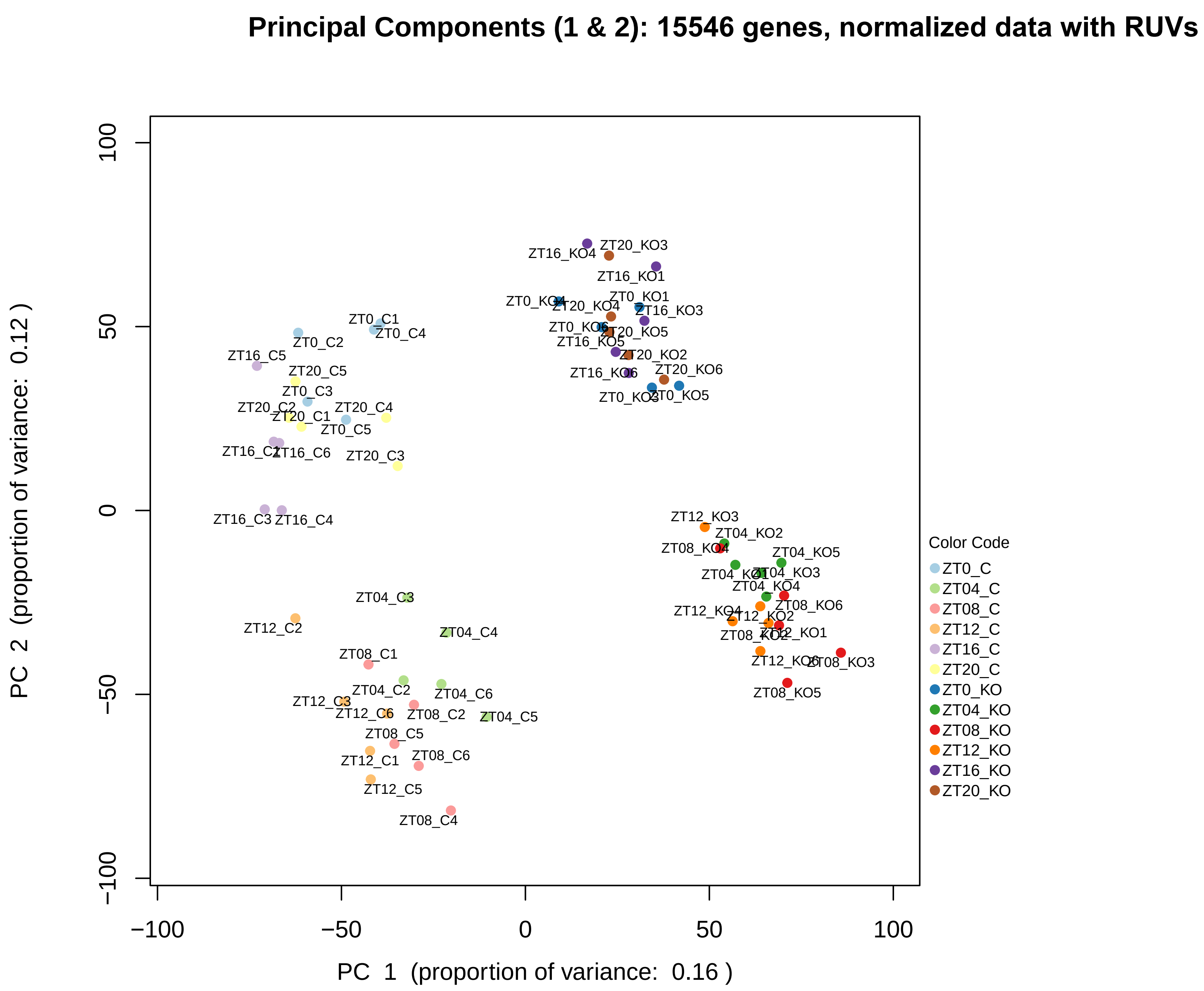

Figure S13: First two principal components of RNA-Seq normalized data with RUVs to remove unwanted variation (k=2).

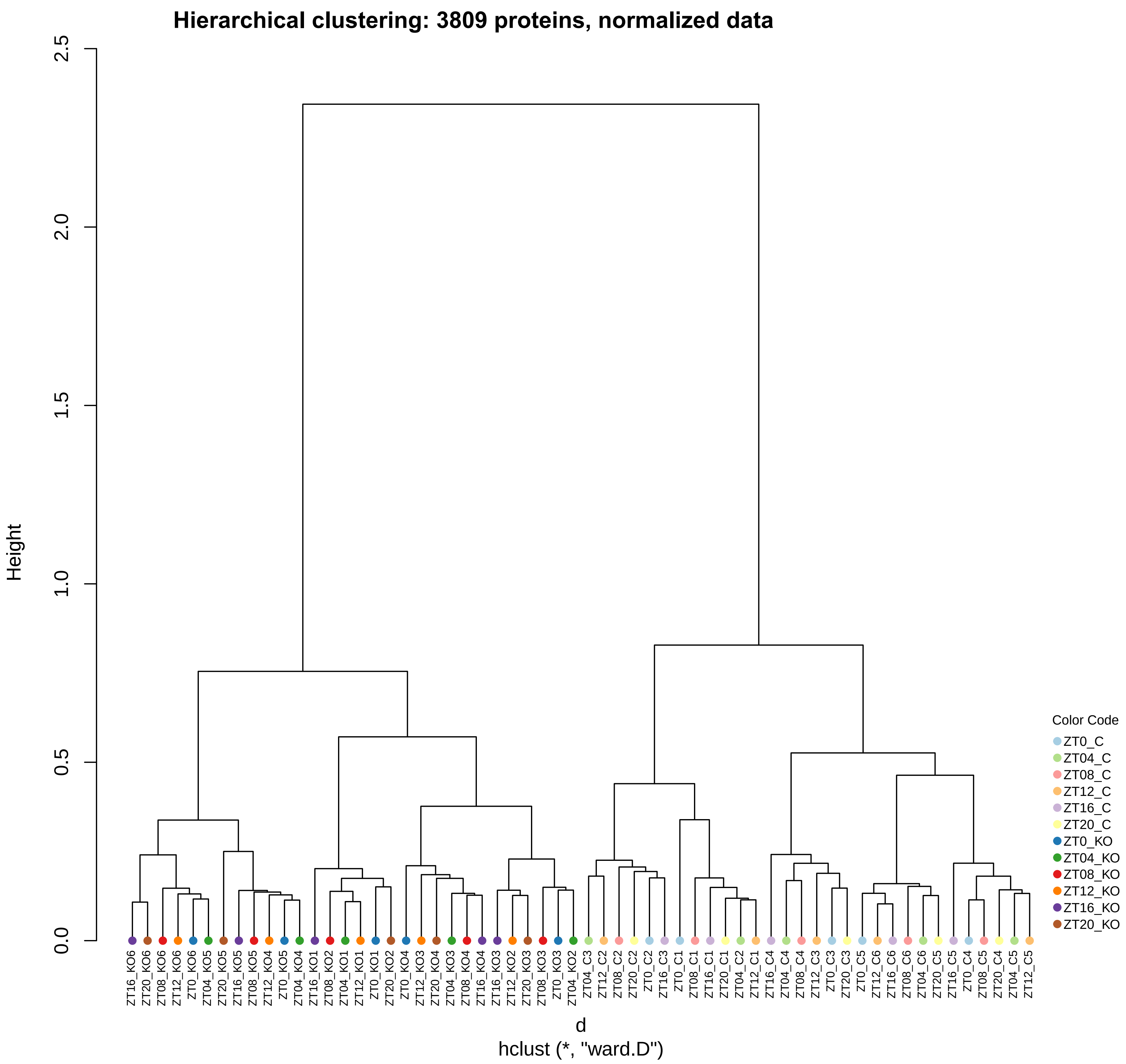

Figure S14: Hierarchical clustering of normalized proteomics data. Proteins were filtered for low expression (value present in at least 80% of samples). In normalized data before RUV treatment, samples cluster by genotype but do not by time point.

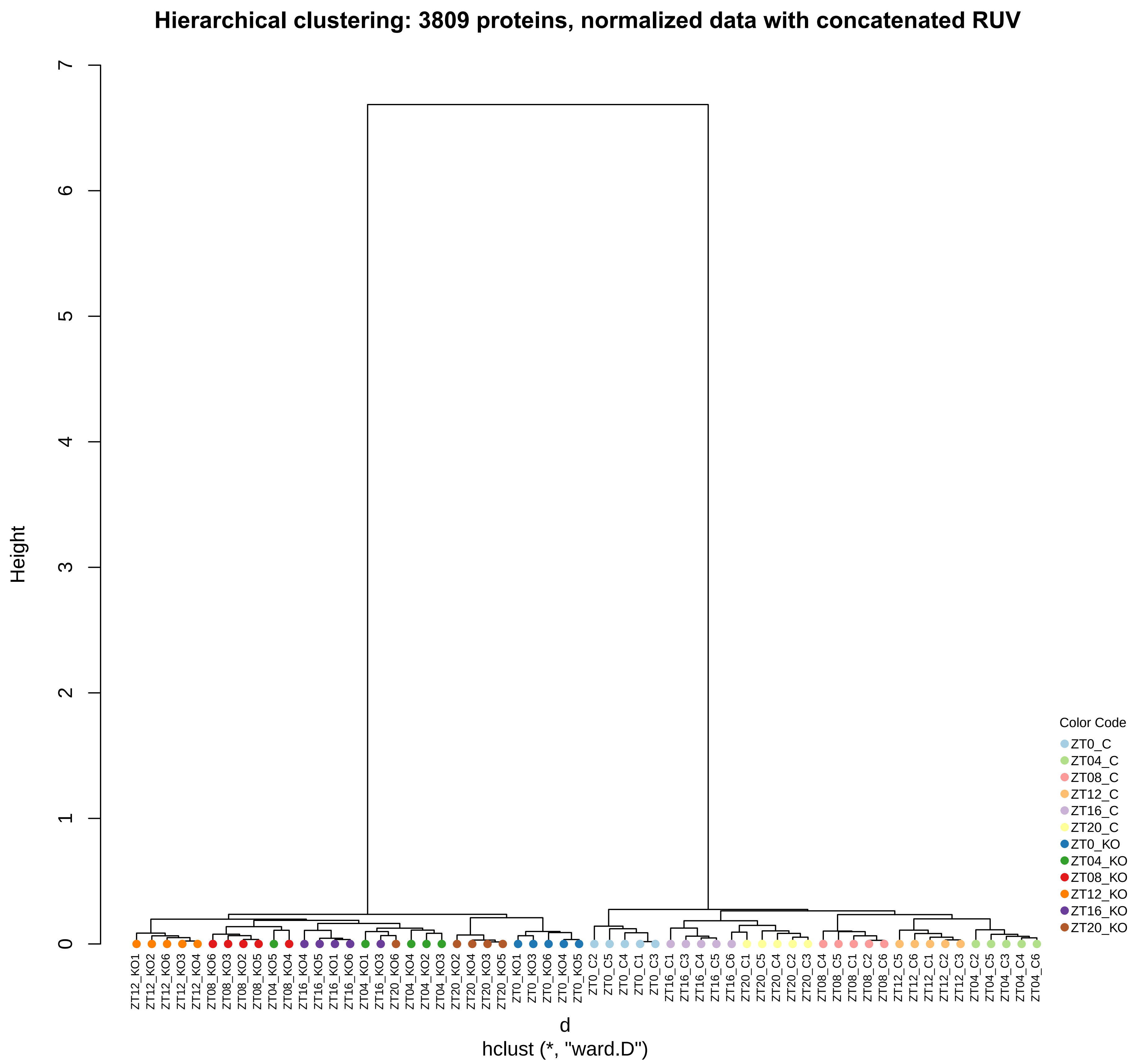

Figure S15: Hierarchical clustering of normalized proteomics data with concatenated RUV. After RUV treatment, samples cluster perfectly by genotype and quite well by time point. Clustering is better in the CTRL genotype than in the cKOt genotype.

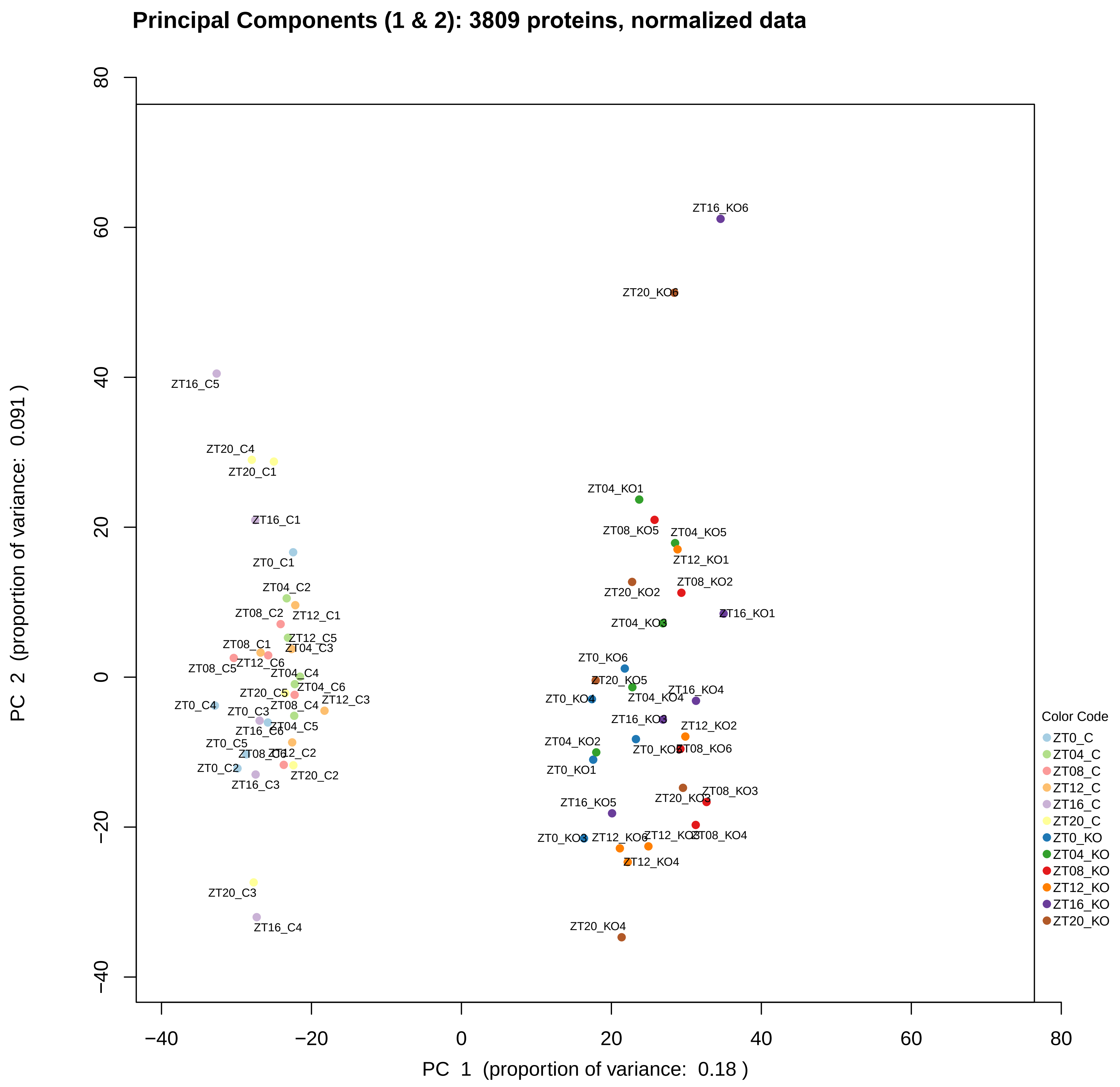

Figure S16: First two principal components of normalized proteomics data.

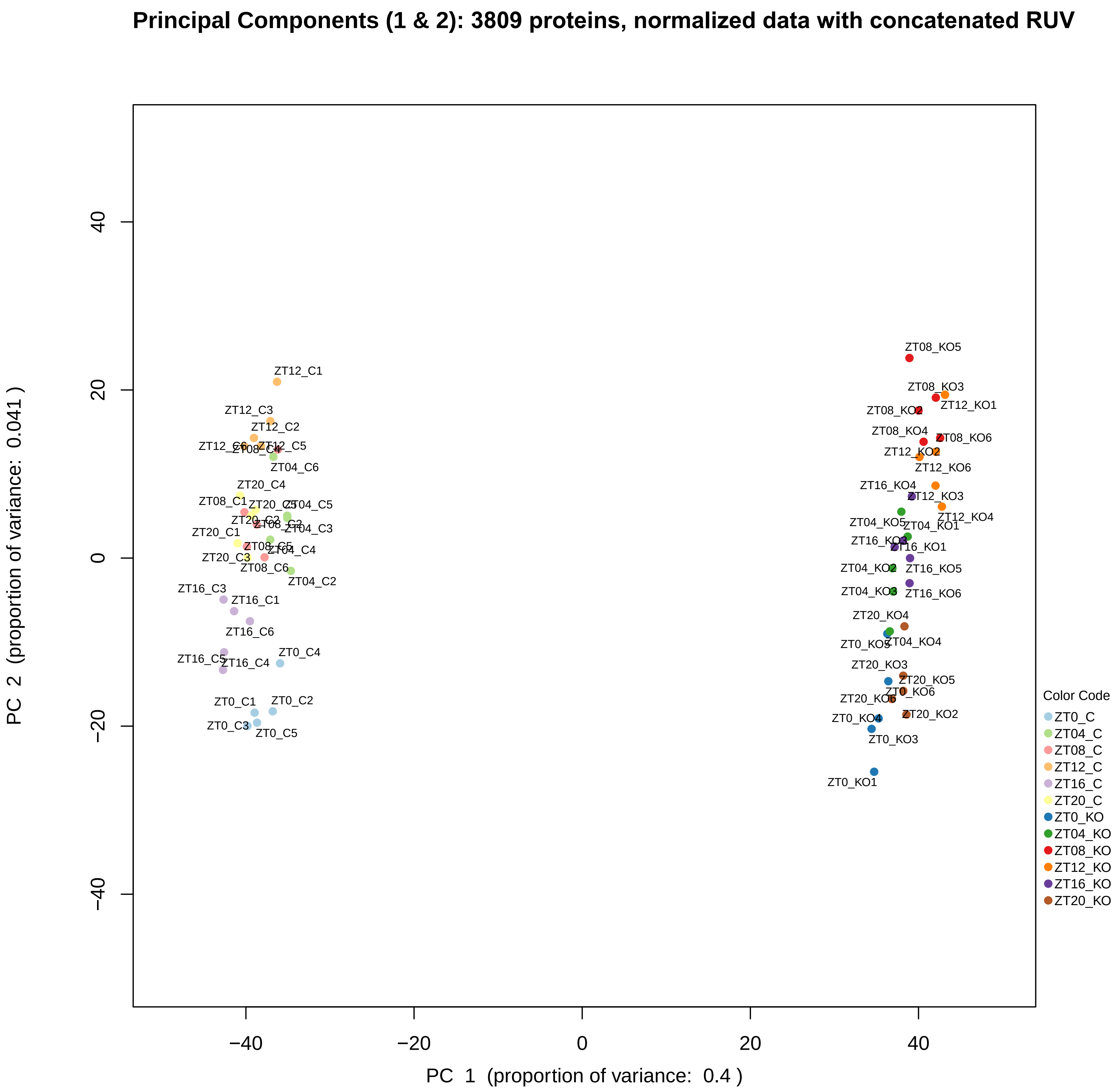

Figure S17: First two principal components of normalized proteomics data with concatenated RUV.

SUPPLEMENTARY METHODS

The SUPPLEMENTARY METHODS is the complete version of the METHODS section.

METHODS

KEY RESOURCES TABLE

RESOURCE AVAILABILITY

- Lead contact
- Materials availability
- Data and code availability

EXPERIMENTAL MODEL AND SUBJECT DETAILS

- Animal studies

METHOD DETAILS

- Production of transcriptomics dataset by RNA-seq
  - *RNA library construction and sequencing*
  - *RNA-Seq reads mapping*
  - *Data processing: normalization and RUV*
  - *Sample selection for analysis*
- Production of proteomics dataset by LC-MS
  - *Tissue extraction for proteome analysis*
  - *Trypsin digestion*
  - *Sample cleanup and fractionation*
  - *LC-MS analysis*
  - *Protein identification and quantification*
  - *Data processing: imputation and RUV*
- Production of metabolomics datasets by LC/MS
  - *Production of renal metabolomics dataset*

Production of plasma metabolomics dataset

Respirometry

STATISTICS: QUANTIFICATION AND STATISTICAL ANALYSIS

Rhythmicity analysis

Comparison of group means

Mapping proteins to genes

KEGG pathway over-representation analysis

STUDY APPROVAL

KEY RESOURCES TABLE (Table S7)

RESOURCE AVAILABILITY

Lead contacts

Further information and requests for reagents and data may be directed to and will be fulfilled by the following lead contacts :

- Yohan Bignon, Department of Biomedical Sciences, University of Lausanne, 27 rue du Bugnon, 1011 Lausanne, Switzerland. e-mail: yohan.bignon@unil.ch
- Dmitri Firsov, Department of Biomedical Sciences, University of Lausanne, 27 rue du Bugnon, 10011 Lausanne, Switzerland. e-mail: dmitri.firsov@unil.ch
- Frédéric Gachon, Institute for Molecular Bioscience, The University of Queensland, Australia. e-mail: f.gachon@uq.edu.au

Materials availability

Further information and request for materials should be directed to and will be fulfilled by lead contacts.

Data and code accessibility.

- All RNAseq raw datasets generated in this work have been deposited into the Gene Expression Omnibus (GEO) database (GSE216252). The mass spectrometry proteomics data have been deposited to the ProteomeXchange Consortium via the PRIDE partner repository (proteomexchange.org) with the dataset identifier PXD036803. The mass spectrometry metabolomics data (two data sets from kidney and plasma, respectively) have been deposited to the Zenodo repository with doi: 10.5281/zenodo.7225427 .
- This paper does not report original code.
- Any additional information required to reanalyze the data reported in this paper is available from lead contacts upon request.

EXPERIMENTAL MODEL AND SUBJECT DETAILS

Animal studies

All experiments were performed on male mice housed under 12-hours light/12-hours dark cycles with *ad libitum* access to drinking water and a standard laboratory chow diet (KLIBA NAFAG diet 3800). Procedures used to generate *Bmal1*$^{lox/lox}$*/Pax8-rtTA*/LC-1 Cre mouse strain (bred on the genetic background of the C57BL/6J mouse from The Jackson Laboratory) were described previously (1). Eight-weeks old *Bmal1*$^{lox/lox}$*/Pax8-rtTA*/LC-1 mice (referred as cKOt mice) and their *Bmal1*$^{lox/lox}$ littermate mice (referred as CTRL or control mice) were treated for 2 weeks with Doxycycline (DOX; 2 mg/ml in drinking water along with 20 mg/ml sucrose) to induce the Cre recombinase inactivation of the *Bmal1* (*Arntl*) gene. This model of tubular core-clock mechanism deletion has been previously described and validated (1). We did not predetermine sample sizes; instead, we selected group sizes based on contemporary work in

the literature and accepted guidelines in the field (2). The investigators were not blinded during experiments.

For production of omics datasets, plasma and kidneys from CTRL and cKOt mice were harvested four weeks after the end of DOX treatment and immediately stored at -80°C. Before freezing, both left and right kidneys were cut transversely into two approximately equal pieces. Two halves of the left kidney were used for transcriptomics and proteomics analyses and a half of the right kidney was used for metabolomics. Blood samples were collected from the tail and centrifuged to produce plasma samples. Afterwhile mice were anesthetized with Ketamine and Xylasine and perfused with phosphate buffer saline through their abdominal aorta, prior to organ collection. A total of 72 mice were used for this purpose: 6 CTRL and 6 cKOt mice, sacrificed at 6 different Zeitgeber time (ZT0, ZT4, ZT8, ZT12, ZT16 and ZT20, with ZT0 and ZT12 corresponding to times when light is switched on and off, respectively).

For additional carnitine measurements, 8-weeks old CTRL and cKOt mice were placed in metabolic cages under *ad libitum* standard diet during the first week of DOX treatment (day 1 to day 7) and after 2 weeks for plasma and urine collection. Mice were then sacrificed 14 days after the end of DOX treatment for liver, heart, brain and skeletal muscle (right rectus femora) collection.

For physical activity experiments, CTRL and cKOt mice were housed 4 weeks after their DOX treatment, either in calorimetry cages linked to a Promethion system recording three dimensions movements every 5 seconds or in circadian cages containing running wheels linked to Clocklab3 software.

For respirometry experiments, mice were treated with DOX and sacrificed 4 weeks later at two circadian time (ZT4 and ZT16). Mice have been perfused with phosphate buffer saline through their abdominal aorta before kidney collection. For each mouse a central cross-section

of the left kidney of about 30 mg has been immediately frozen at -80°C prior to respirometry analysis.

METHOD DETAILS

Production of transcriptomics dataset by RNA-seq

RNA library construction and sequencing

RNA from frozen half-kidneys of 72 mice were extracted and purified using RNAeasy MiniElute Spin Column (Qiagen). RNA quality was assessed on a Fragment Analyzer (Agilent Technologies). All RNAs had an RQN between 7.5 and 9.7. RNA-seq libraries were prepared from 200 ng of total RNA with the Illumina TruSeq Stranded mRNA reagents (Illumina) using a unique dual indexing strategy, and following the official protocol automated on a Sciclone liquid handling robot (PerkinElmer). Libraries were quantified by a fluorometric method (QubIT, Life Technologies) and their quality assessed on a Fragment Analyzer (Agilent Technologies). Clusters were generated with 2 nM of an equimolar pool from the resulting libraries using the Illumina HiSeq 3000/4000 SR Cluster Kit reagents. Sequencing was performed on the Illumina HiSeq 4000 using HiSeq 3000/4000 SBS Kit reagents for 150 cycles (single read). Sequencing data were demultiplexed, filtered for failed reads, and written to FASTQ files using the bcl2fastq2 conversion software (version 2.20, Illumina).

RNA-Seq reads mapping

Sequence reads in FASTQ files were processed as follows: Low-quality 3' ends and adapters were trimmed off using Cutadapt (version 1.8) [https://doi.org/10.14806/ej.17.1.200]. Reads mapping to ribosomes were removed with fastq_screen (version 0.11.1) (3). Remaining reads were filtered for low complexity with reaper (version 15-065) (4). The reads that passed these data cleaning steps were aligned against the *Mus musculus*. GRCm38.98 genome using STAR

(version 2.5.3a) (5). The number of read counts per gene locus was obtained with htseq-count (version 0.9.1) (6).

Data processing: normalization and RUV

Further data processing was performed in R (version 4.0.3). Raw counts were transformed to counts per million (CPM), and genes with a low number of counts were filtered out according to the following rule: at least one sample in the whole data set had to have at least 1 CPM reads for a gene to be retained in the data set. Library sizes were then scaled using TMM normalization. Subsequently, the normalized counts were transformed to CPM values and a log2 transformation was applied using the R Bioconductor package edgeR (7).

Data was corrected for unwanted variation by the method RUVs from the R Bioconductor package RUVSeq (8). This R package offers a family of normalization methods that correct for complex unwanted technical effects of unknown origin or not aligned with the experimental design. They are based on the "Remove Unwanted Variation" (RUV) strategy developed in (9) and (Gagnon-Bartsch J, Jacob L and Speed TP (2013) Removing unwanted variation from high dimensional data with negative controls Technical Report 820, Dept. Statistics, Univ. California, Berkeley, Berkeley, CA. https://statistics.berkeley.edu/tech-reports/820) and adapted for RNA-Seq data. The RUVs method, specifically, uses factor analysis on the differences between replicate groups of samples for estimating factors of unwanted variation, which can then be included in a linear model. As for parameter settings in the current analysis, the number of factors of unwanted variation estimated was two (k=2), and all genes in the normalized data set were used as control genes (default setting for control genes). A quality check by hierarchical clustering and by plotting of the two first principal components confirmed that sample clustering into replicate groups improved after applying the RUVs method with these parameters. The two genotypes are well separated; Time points of the light phase until the onset of darkness cluster together (ZT04, ZT08 and ZT12) as do the

time points of the dark period until the light switches on (ZT16, ZT20, ZT0). These quality control plots are provided for raw data, normalized data before RUVs treatment, and normalized data after RUVs treatment as supplementary figures S8 through S13.

For the subsequent statistical analysis of rhythmic patterns using the R package dryR **(see below, section "Rhythmicity analysis")**, RUVs was applied to normalized logCPM data and the corrected data table was used as input. For differential expression analysis using the R package limma (10) **(see below, section "Comparison of group means")**, two parameters estimated by RUVs were included as covariates in the linear model, while the normalized logCPM data without RUVs correction was used as expression matrix input, as is recommended by the authors of the RUVSeq package for this type of analysis.

Sample selection for analysis

From six biological replicate samples that were available per time point and genotype, five were selected for further data analysis, which allowed the removal of outliers and reduced the size of the RNA-Seq data set from 72 to 60 samples (6 time points x 2 genotypes x 5 replicates). Selection criteria were the RNA integrity number (RIN) from the Agilent Bioanalyzer system, which measures RNA quality, and distance from the other replicate samples in principal component analysis (PCA) or multi-dimensional scaling (MDS) plots. Table ST8 displays decision criteria and RIN values for all 72 mice. The same 60 individual mice thus chosen were then also used to procure samples for proteomics and metabolomics analyses in order to have matching data sets.

Production of proteomics dataset by LC-MS

Kidney proteomics dataset has been produced using 60 half-kidneys samples from CTRL and cKOt mice sacrificed at 6 different circadian times. The 60 samples were processed in 5 batches

of 12 (each time 6 WT, 6 cKOt), with each batch corresponding to a full time series for each genotype.

Tissue extraction for proteome analysis

Frozen half kidneys were ground in a 15x excess (v:w) of 80% (v/v) methanol chilled at -20C, using a Fastprep system and ceramic beads. Tubes were shaken twice for 30 s at maximum speed, with chilling on ice for 5 min between treatments. Tubes were then kept at -20C for 1h, after which they were centrifuged at 3000 g for 5 min and the methanol supernatant was removed. Tissue samples and beads were allowed to dry with open lid for 5 min under a laminar flow. A 10x amount (v:w, e.g., 1.0 ml for 100mg of tissue) of miST buffer (1% sodium deoxycholate, 30 mM Tris pH 8.6, 10 mM DTT) was added and tubes were shaken again on the FastPrep for 2x 30s as for the previous step. 500 µl of each crude homogenate were diluted 1:1 with miST buffer and heated at 95°C for 10min. The protein concentration of the resulting sample was determined by the tryptophane fluorescence method (11) and samples were frozen in aliquots of 100 µg, adjusted at 2 µg/µl with miST buffer.

SILAC-labelled mouse kidneys were obtained by feeding mice a diet containing heavy isotope labelled Lysine (13C6-Lys) as described by Mauvoisin et al. (12) and Krüger et al. (13). SILAC mouse tissues were handled in the same manner up to storage. An equimolar pool of tissues from SILAC mouse ZT=0 and ZT=12h was prepared, adjusted to 2 µg/µl with miST buffer and used as reference SILAC heavy isotope sample. An aliquot of SILAC heavy isotope labelled tissue extract was mixed 1:1 with each kidney extract before digestion and used as unique internal reference to which ratios were calculated for all samples.

Trypsin digestion

Aliquots of samples were digested following a modified version of the iST method (14). Thawed tissue extracts were vortexed thoroughly. 50 µl (=100 µg) of each sample were mixed 1:1 with the heavy SILAC reference sample, boiled at 95°C for 5 min and diluted 1:1 with

water. Chloroacetamide was added to reach a final concentration of 40 mM followed by incubation at RT for 45 min in the dark to alkylate Cys residues. Samples were adjusted to 3 mM EDTA final and digested by adding Trypsin/LysC (Promega, Prod.Nr. V5072) at a ratio of 1:100 and incubating for 1 h at 37°C with gentle shaking. A second digestion step was performed by adding the same amount of enzymes (final 50:1 ratio) and incubating for 2 h at 37°C.

Sample cleanup and fractionation

Half of each digest (=100 µg) was processed further by adding 2 volumes of 1%TFA in 100% isopropanol and loading onto a previously conditioned Oasis MCX SPE microplate (Waters Corp., Milford, MA, prod.#186001830BA). Wells were washed 3x by centrifugation with 500 µl Isopropanol, 1% TFA and 1x with 2% MeCN, 0.05% TFA. Elution was performed to obtain three fractions per sample, with 200ul of 1) 75 mM Ammonium acetate in 50% MeCN (pH 4), 2) 150 mM Ammonium acetate in 50% MeCN (pH 5.6) and 3) 1% NH3 in 80% MeCN/19% water (pH>10), respectively. All eluates were frozen in liquid nitrogen and dried by evaporation.

LC-MS analysis

Dried fractions were resuspended in 60 µl of 2% MeCN, 0.05% TFA. 5 µl of each sample were analysed on an Orbitrap Fusion Tribrid mass spectrometer (Thermo Fisher Scientific) interfaced through a nano-electrospray ion source to an Ultimate 3000 RSLCnano HPLC system (Thermo Fisher Scientific). Peptides were separated on a reversed-phase custom packed 40 cm C18 column (75 µm ID, 100Å, Reprosil Pur 1.9 µm particles) with a 4-76% (v/v) acetonitrile gradient in 0.1% (v/v) formic acid (140 minutes gradient). Full MS survey scans were performed at 120,000 resolution. A data-dependent acquisition (DDA) method, controlled by Xcalibur software, was used to select precursors in "top speed" mode with a cycle time of 0.6 s. Masses were isolated with a window of 1.6 m/z, fragmentation done in HCD mode with

32% energy, and fragments analyzed in the ion trap. Peptides selected for MS/MS were excluded from further fragmentation during 60 s.

Protein identification and quantification

Raw MS data were processed by the MaxQuant software (version 1.6.14.0) integrating the Andromeda search engine (15). The SWISSPROT mouse proteome database of September 19th, 2020 including validated splice variants was used (25'321 sequences), with sequences of common contaminants added. False discovery rate filtering of both peptide spectrum matches (PSM) and protein identifications was fixed at 1%. Search parameters allowed for 2 missed cleavages and protease specificity was set to trypsin (K, R) with cleavage after prolines included. Carbamidomethyl on cysteines was set as fixed modification, and acetyl at the protein N-terminal and oxidation on methionines as variable modification. The SILAC-labeled kidneys were frozen at -80°C until time of extraction. Quantitation of individual protein intensities relative to the reference was performed by MaxQuant as described (15) and was based on the median ratio of peptides for each protein. Global normalization of total protein intensities relative to the heavy standard in each heavy:light mix (to correct for uneven mixing ratios) was also performed automatically by the software as part of the standard MaxQuant workflow for SILAC quantitation (15). Initial mass precursor tolerance was 20 ppm and was then dynamically adjusted to 5-6 ppm by MaxQuant after recalibration, and fragment mass tolerance was fixed at 0.5 Da. The MaxQuant output file proteinGroups.txt was further processed with the Perseus software (16). We used SILAC ratios normalized internally by MaxQuant for all further analyses. Proteins only identified by modified peptides, reverse hits and known contaminants were eliminated and all SILAC ratios were log2 transformed. The resulting raw table contained 6,993 protein groups.

Data processing: imputation and RUV

Further data processing performed in R (version 4.1.0). The proteomics data set had 34% missing data values. Proteins with fewer than 48 out of 60 data values (3164 features) were removed from the data set prior to statistical analysis. Also, proteins that did not have at least two peptides used in quantification (data column "razor+unique" < 2) were removed (272 features). The resulting filtered data table contained 3809 features. The missing values in this filtered data table were imputed with the R package missForest with default settings (17), using a random forest trained on the available data values to predict the missing data points.

An RUV normalization was applied to the proteomics data to correct for batch effect and unwanted variation of unknown sources. All RUV correction steps described hereafter were performed with the method RUVIII from the R package ruv, which relies on replicate groups like the method RUVs that was used for RNA-Seq data. We used all features as negative control features.

We employed a hierarchical approach similar to the inter-batch correction strategy presented in (18), which was implemented in the R package hRUV and was developed for large omics data sets with batch effect. This publication introduced the concept of sequential batch correction: Instead of treating a large data set all at once for batch correction, one can start with a sub-set of the data and correct only this, then add more and more batches sequentially for several correction rounds, which allows to dynamically change normalization factors from round to round. The authors propose two tree-structured approaches (balanced and concatenating) for sequential, hierarchical merging of batches. We used a mix of the two types of structures in a two-level strategy. In a first step, the data was divided into cKOt and control samples subsets and a concatenation strategy was applied to the samples from each genotype separately. There were five batches of six samples from each genotype. We started with three batches to create a starting data set large enough for an RUV normalization and applied RUVIII to it, then added the fourth batch and ran RUVIII again, then included the fifth batch and ran

RUVIII once more. The number of factors of variation to estimate was set to k=3, k=4 and k=5, respectively, in the three rounds of RUVIII (number of batches that were included in each round). In a second step, we combined the thus corrected data from the two genotypes and performed RUVIII for a final correction, with k=2. We conceive of this step as a data merging procedure with a simple balanced structure. As a quality control and aid in fine-tuning the details of our approach, hierarchical clustering, plots of the first two principal components and RLE plots were generated and used to visually assess improvement in sample clustering and reduction in variability. After completion of this two-level RUVIII-based normalization, the initially imputed values were removed, then re-imputed using the now-normalized data. Plots of hierarchical clustering and of principal components before RUV treatment and after RUV treatment with re-imputation are provided in supplementary figures S14 through S17.

Production of metabolomics datasets by LC/MS

Production of renal metabolomics dataset

Kidney and plasma metabolomics datasets have been produced using 60 half-kidneys samples or 60 plasma samples, from CTRL and cKOt mice sacrificed at 6 different circadian times.

Kidney metabolomics data have been produced by the Metabolon company according to its standard methods described below. Samples were prepared using the automated MicroLab STAR® system from Hamilton Company. Several recovery standards were added prior to the first step in the extraction process for QC purposes. To remove protein, dissociate small molecules bound to protein or trapped in the precipitated protein matrix, and to recover chemically diverse metabolites, proteins were precipitated with methanol under vigorous shaking for 2 min (GenoGrinder 2000; Glen Mills Inc) followed by centrifugation. The resulting extract was divided into five fractions: two for analysis by two separate reverse phase (RP)/UPLC-MS/MS methods with positive ion mode electrospray ionization (ESI), one for

analysis by RP/UPLC-MS/MS with negative ion mode ESI, one for analysis by HILIC/UPLC-MS/MS with negative ion mode ESI, and one sample was reserved for backup. Samples were placed briefly on a TurboVap® (Zymark) to remove the organic solvent. Sample extracts were stored overnight under nitrogen before preparation for analysis. Several types of controls were analyzed in concert with the experimental samples: a pooled matrix sample generated by taking a small volume of each experimental sample (or alternatively, use of a pool of well-characterized human plasma) served as a technical replicate throughout the data set; extracted water samples served as process blanks; and a cocktail of QC standards that were carefully chosen not to interfere with the measurement of endogenous compounds were spiked into every analyzed sample, allowed instrument performance monitoring and aided chromatographic alignment. Experimental samples were randomized across the platform run with QC samples spaced evenly among the injections in mass spectrometer.

All methods utilized a Waters ACQUITY ultra-performance liquid chromatography (UPLC) and a Thermo Scientific Q-Exactive high resolution/accurate mass spectrometer interfaced with a heated electrospray ionization (HESI-II) source and Orbitrap mass analyzer operated at 35,000 mass resolution. The sample extract was dried then reconstituted in solvents compatible to each of the four methods. Each reconstitution solvent contained a series of standards at fixed concentrations to ensure injection and chromatographic consistency. One aliquot was analyzed using acidic positive ion conditions, chromatographically optimized for more hydrophilic compounds. In this method, the extract was gradient eluted from a C18 column (Waters UPLC BEH C18-2.1x100 mm, 1.7 µm) using water and methanol, containing 0.05% perfluoropentanoic acid (PFPA) and 0.1% formic acid (FA). Another aliquot was also analyzed using acidic positive ion conditions; however it was chromatographically optimized for more hydrophobic compounds. In this method, the extract was gradient eluted from the same afore mentioned C18 column using methanol, acetonitrile, water, 0.05% PFPA and 0.01%

FA and was operated at an overall higher organic content. Another aliquot was analyzed using basic negative ion optimized conditions using a separate dedicated C18 column. The basic extracts were gradient eluted from the column using methanol and water, however with 6.5mM Ammonium Bicarbonate at pH 8. The fourth aliquot was analyzed via negative ionization following elution from a HILIC column (Waters UPLC BEH Amide 2.1x150 mm, 1.7 µm) using a gradient consisting of water and acetonitrile with 10mM Ammonium Formate, pH 10.8.

The MS analysis alternated between MS and data-dependent MSn scans using dynamic exclusion. The scan range varied slighted between methods but covered 70-1000 m/z. Raw data was extracted, peak-identified and QC processed using Metabolon's hardware and software. These systems are built on a web-service platform utilizing Microsoft's .NET technologies, which run on high-performance application servers and fiber-channel storage arrays in clusters to provide active failover and load-balancing. Compounds were identified by comparison to library entries of purified standards or recurrent unknown entities. Metabolon maintains a library based on authenticated standards that contains the retention time/index (RI), mass to charge ratio (m/z), and chromatographic data (including MS/MS spectral data) on all molecules present in the library. Furthermore, biochemical identifications are based on three criteria: retention index within a narrow RI window of the proposed identification, accurate mass match to the library +/- 10 ppm, and the MS/MS forward and reverse scores between the experimental data and authentic standards. The MS/MS scores are based on a comparison of the ions present in the experimental spectrum to the ions present in the library spectrum. While there may be similarities between these molecules based on one of these factors, the use of all three data points can be utilized to distinguish and differentiate biochemicals. More than 3300 commercially available purified standard compounds have been acquired and registered into LIMS for analysis on all platforms for determination of their analytical characteristics. Additional mass spectral entries have been created for structurally

unnamed biochemicals, which have been identified by virtue of their recurrent nature (both chromatographic and mass spectral). These compounds have the potential to be identified by future acquisition of a matching purified standard or by classical structural analysis. A variety of curation procedures were carried out to ensure that a high-quality data set was made available for statistical analysis and data interpretation. The QC and curation processes were designed to ensure accurate and consistent identification of true chemical entities, and to remove those representing system artifacts, mis-assignments, and background noise. Metabolon data analysts use proprietary visualization and interpretation software to confirm the consistency of peak identification among the various samples. Library matches for each compound were checked for each sample and corrected if necessary. Peaks were quantified using area-under-the-curve. A data normalization step was performed to correct variation resulting from instrument inter-day tuning differences. Essentially, each compound was corrected in run-day blocks by registering the medians to equal one (1.00) and normalizing each data point proportionately.

Before statistical analysis, metabolites with more than 20% missing data values across all samples were removed from the data set. The remaining missing values were imputed with the R package missForest with default parameters. Data was treated for with glog2 from the R package MKmisc for variance stabilization, and as a final processing step, data imputation with missForest was re-computed on the thus transformed data. The data table used for statistical analysis contained 814 metabolites.

Production of plasma metabolomics dataset

Plasma metabolomics data have been produced by the Biocrates company according to its standard operating procedures and state-of-the-art techniques, using the MxP® Quant 500 kit. Measurements have been done using proprietary sample preparation and MS/MS analytical methods (MRM, precursor scans and neutral loss scans) for targeted classes of metabolites.

Mass chromatograms have been interpreted using Biocrates software and third-party softwares. Data were log2 transformed. Metabolites with missing data values were removed from the data set, making data imputation unnecessary. The data table used for statistical analyses contained 332 metabolites plus a metabolite ratio (kynurenine/tryptophan).

Respirometry

Central renal cross-sections (containing cortex, outer medulla and inner medulla) from 4 CTRL and 5 cKOt mice at ZT4 and 7 CTRL and 5 cKOt mice at ZT16 were processed in sextuplets. Tissue homogenization and respirometry in frozen samples (RIFS) were adapted from a previously published protocol (19). ~30 mg of frozen kidney slice were thawed in ice-cold PBS, minced, and homogenized in MAS (70 mM sucrose, 220 mM mannitol, 5 mM KH_2PO_4, 5 mM $MgCl_2$, 1 mM EGTA, 2 mM HEPES pH 7.4). Each sample was mechanically homogenized with 15 strokes in glass–glass Dounce homogenizer (Wheaton Glass tissue grinder, Fisher Scientific). Homogenates were centrifuged at 1,000 g for 10 min at 4°C; then, the supernatant was collected to assess protein concentration using BCA protein assay kit. 2.5 µg of homogenates was loaded into Seahorse XFe96 microplate in 20 µl of MAS. The loaded plate was centrifuged at 2,000 g for 5 min at 4°C (no brake), and an additional 130 µl of MAS containing cytochrome c (10 µg/ml, final concentration) was added to each well. Substrate injection was as follows: NADH (2 mM) or 5 mM succinate + rotenone (5 mM + 2 µM) were injected at port A; rotenone + antimycin A (2 µM + 4 µM) at port B; TMPD + ascorbic acid (0.5 mM + 1 mM) at port C; and azide (50 mM) at port D. These conditions allow for the determination of the respiratory capacity of mitochondria through Complex I, Complex II, and Complex IV. The experiment was performed at 37°C.

STATISTICS: QUANTIFICATION AND STATISTICAL ANALYSIS

Differential Rhythmicity analysis

Analysis of rhythmic patterns was performed using the R package *dryR* [(10); **https://github.com/naef-lab/dryR]**. *dryR* is performs differential rhythmicity of omics datasets with two (or more) sample groups. The present study represents a two-group design (i.e., two genotypes CTRL and cKO), in this scenario *dryR* fits five models to each feature and selects a model using the Bayesian information criterion (BIC): model 1: no rhythmicity in either group; model 2: rhythmic in CTRL, non-rhythmic in cKO (loss-of-rhythm); model 3: non-rhythmic in CTRL, rhythmic in cKO (gain-of rhythm); model 4: rhythmic in both groups with identical acrophase and amplitude (unaltered rhythm); model 5: rhythmic in both groups with differing acrophase or amplitude between groups (altered rhythm).

For all data sets including RNA-Seq, proteomics, metabolomics in kidney and metabolomics in plasma, pre-processed normalized data were analysed using *dryR*'s drylm function. This function expect normally distributed data and internally uses the base R function lm for fitting the sinusoidal curves.

Internal to drylm, a model selection method, based on the BIC, is employed to determine the best-fitting model for each feature. Starting from the BICs from all five models, Schwartz weights (BICW) are calculated for the models, and the one with the greatest BICW is retained. The BICWs give an indication of how well a model's BIC is distinguished from the lowest BIC among the five models. The five BICWs are used as a measure of confidence in the model that was selected for a particular feature. A threshold can be applied to the BICWs to separate out features with low-confidence model assignment. In the present study, features with BICW < 0.65 for the best-fitting model were considered not classifiable ("ambiguous" model).

Comparison of group means

Differential gene expression as well as differential abundance of proteins and metabolites were assessed by computing moderated t-statistics with the R Bioconductor package limma, comparing the group means of cKOt and control mice (30 versus 30 mice). For genes, the approach limma-trend was used, as described and recommended for RNA-Seq data in (20), with parameter setting trend=TRUE in the function eBayes (in estimating the variance between samples by the eBayes approach, this setting allows for a trend in the prior that is dependent on expression values and serves to take into account mean-variance relationship). For proteomics and metabolomics data, the default parameter setting trend=FALSE was retained (for a constant prior of the variance used globally in the whole data set.) P-values from this group comparison were adjusted for multiple testing by the Benjamini-Hochberg method, which controls for the false discovery rate (FDR).

Covariates were included in the statistical model's design for two purposes. Firstly, circadian rhythmic oscillations, which were found in many of the molecules but were not of direct interest in the comparison of means, needed to be adjusted for. Two covariates were therefore included to fit a sine and a cosine curve, both conceived to start at time ZT0 (with value 0 and 1, respectively) and to cover a 24 h period. In detail: The variable for the sine curve was set to a value determined by the time point of sacrifice : ZT0=0, ZT4=0.866, ZT8=0.866, ZT12=0, ZT16=-0.866, ZT20=-0.866. Similarly, the variable for the cosine curve was set to the following values: ZT0=1, ZT4=0.5, ZT8=-0.5, ZT12=-1, ZT16=-0.5, ZT20=0.5. The comparison of group means thus served to estimate the effect of genotype on gene expression (or protein or metabolite abundance) that was independent of variations in sinusoidal rhythmic patterns. Secondly, in the RNA-Seq data only, two more covariates from an RUV analysis were added to correct for unwanted technical variation between samples, as mentioned in the section on RNA-Seq data processing. P-values and adjusted p-values reported are from these models with covariates. On the other hand, log fold changes reported are simply the log2 ratios of

arithmetic group means across all time points and thus do not reflect the full complexity of the linear models.

Mapping of proteins to genes

The results presented in Fig. 2A-D required pairing of protein features in the proteomics data set to the corresponding coding genes in the RNA-Seq data set. We started from the filtered proteomics data table prepared as described in the section *Data processing: imputation and RUV*. Gene names from the proteomics data table (provided by the MaxQuant software) were mapped to gene symbols in RNA-Seq data table (from the Gencode annotation GRCm38.98). Aliases were resolved manually using the NCBI Gene online portal at https://www.ncbi.nlm.nih.gov/gene. If a protein was not uniquely identifiable from the mass spectrometry peak and was mapped to two or more gene names by MaxQuant, all mappings were retained as separate protein-gene pairs. Protein features with no gene name given by MaxQuant, or with a gene name not present in the Gencode annotation, were removed. Protein-gene pairs where the gene had been filtered out of our data before analysis because of low read count were removed. This resulted in 3810 protein-gene pairs that could be used for analysis.

KEGG pathway over-representation analysis

Over-representation analysis (ORA) of genes from KEGG pathways (21) was performed with the R package clusterProfiler (22, 23) using a one-sided Fisher's exact test. The function bitr was used to convert NCBI gene symbols, which were available in the gene annotation, to Entrez IDs (24), which were available in the KEGG database; genes that could not be mapped and were dropped from the analysis. Out of 15543 gene symbols submitted to ID conversion, 96% were found in KEGG. ORA requires an input gene list: generally, a list of differentially expressed genes from a comparison of interest, and will test whether genes from each pathway

are present in this list in higher numbers than expected by chance. Our list contained 2,265 genes (after successful mapping to KEGG) that were differentially expressed between cKOt and control mice with adjusted p-value < 0.05 and absolute fold change > 1.2 (values from limma analysis). ORA also needs a definition of the so-called "universe" or "background" of all genes being considered. Following the recommendation in (25), the background list for the ORA consisted of all transcripts that were found to be expressed in renal tubules, i.e., that were present with annotation in the RNA-Seq data set and had passed initial filtering for low counts, and its size was 14947 genes (after successful mapping to KEGG).

For proteins, the same method with the same thresholds on adjusted p-values and fold changes was applied. The input list consisted of 959 differentially expressed proteins and the background list of 3803 proteins, all of which were successfully mapped to Entrez IDs in the KEGG data base. (KEGG version for both analyses: download from April 29, 2022).

STUDY APPROVAL

Experiments with animals were performed in accordance with the Swiss guidelines for animal care, which conform to the National Institutes of Health animal care guidelines and approved by Swiss cantonal (Canton de Vaud) and federal veterinary authorities (authorization #3488 to DF).

1. Nikolaeva S, Ansermet C, Centeno G, Pradervand S, Bize V, Mordasini D, et al. Nephron-Specific Deletion of Circadian Clock Gene Bmal1 Alters the Plasma and Renal Metabolome and Impairs Drug Disposition. *J Am Soc Nephrol.* 2016;27(10):2997-3004.
2. Hughes ME, Abruzzi KC, Allada R, Anafi R, Arpat AB, Asher G, et al. Guidelines for Genome-Scale Analysis of Biological Rhythms. *Journal of biological rhythms.* 2017;32(5):380-93.
3. Wingett SW, and Andrews S. FastQ Screen: A tool for multi-genome mapping and quality control. *F1000Res.* 2018;7:1338.
4. Davis MP, van Dongen S, Abreu-Goodger C, Bartonicek N, and Enright AJ. Kraken: a set of tools for quality control and analysis of high-throughput sequence data. *Methods (San Diego, Calif).* 2013;63(1):41-9.

5. Dobin A, Davis CA, Schlesinger F, Drenkow J, Zaleski C, Jha S, et al. STAR: ultrafast universal RNA-seq aligner. *Bioinformatics.* 2013;29(1):15-21.
6. Anders S, Pyl PT, and Huber W. HTSeq--a Python framework to work with high-throughput sequencing data. *Bioinformatics.* 2015;31(2):166-9.
7. Robinson MD, McCarthy DJ, and Smyth GK. edgeR: a Bioconductor package for differential expression analysis of digital gene expression data. *Bioinformatics.* 2010;26(1):139-40.
8. Risso D, Ngai J, Speed TP, and Dudoit S. Normalization of RNA-seq data using factor analysis of control genes or samples. *Nat Biotechnol.* 2014;32(9):896-902.
9. Gagnon-Bartsch JA, and Speed TP. Using control genes to correct for unwanted variation in microarray data. *Biostatistics.* 2012;13(3):539-52.
10. Weger BD, Gobet C, David FPA, Atger F, Martin E, Phillips NE, et al. Systematic analysis of differential rhythmic liver gene expression mediated by the circadian clock and feeding rhythms. *Proc Natl Acad Sci U S A.* 2021;118(3).
11. Wiśniewski JR, and Gaugaz FZ. Fast and sensitive total protein and Peptide assays for proteomic analysis. *Anal Chem.* 2015;87(8):4110-6.
12. Mauvoisin D, Wang J, Jouffe C, Martin E, Atger F, Waridel P, et al. Circadian clock-dependent and -independent rhythmic proteomes implement distinct diurnal functions in mouse liver. *Proc Natl Acad Sci U S A.* 2014;111(1):167-72.
13. Krüger M, Moser M, Ussar S, Thievessen I, Luber CA, Forner F, et al. SILAC mouse for quantitative proteomics uncovers kindlin-3 as an essential factor for red blood cell function. *Cell.* 2008;134(2):353-64.
14. Kulak NA, Pichler G, Paron I, Nagaraj N, and Mann M. Minimal, encapsulated proteomic-sample processing applied to copy-number estimation in eukaryotic cells. *Nat Methods.* 2014;11(3):319-24.
15. Cox J, and Mann M. MaxQuant enables high peptide identification rates, individualized p.p.b.-range mass accuracies and proteome-wide protein quantification. *Nat Biotechnol.* 2008;26(12):1367-72.
16. Tyanova S, Temu T, Sinitcyn P, Carlson A, Hein MY, Geiger T, et al. The Perseus computational platform for comprehensive analysis of (prote)omics data. *Nat Methods.* 2016;13(9):731-40.
17. Stekhoven DJ, and Bühlmann P. MissForest--non-parametric missing value imputation for mixed-type data. *Bioinformatics.* 2012;28(1):112-8.
18. Kim T, Tang O, Vernon ST, Kott KA, Koay YC, Park J, et al. A hierarchical approach to removal of unwanted variation for large-scale metabolomics data. *Nature communications.* 2021;12(1):4992.
19. Acin-Perez R, Benador IY, Petcherski A, Veliova M, Benavides GA, Lagarrigue S, et al. A novel approach to measure mitochondrial respiration in frozen biological samples. *Embo j.* 2020;39(13):e104073.
20. Law CW, Chen Y, Shi W, and Smyth GK. voom: Precision weights unlock linear model analysis tools for RNA-seq read counts. *Genome Biol.* 2014;15(2):R29.
21. Kanehisa M, and Goto S. KEGG: kyoto encyclopedia of genes and genomes. *Nucleic Acids Res.* 2000;28(1):27-30.
22. Yu G, Wang LG, Han Y, and He QY. clusterProfiler: an R package for comparing biological themes among gene clusters. *Omics.* 2012;16(5):284-7.
23. Wu T, Hu E, Xu S, Chen M, Guo P, Dai Z, et al. clusterProfiler 4.0: A universal enrichment tool for interpreting omics data. *Innovation (Camb).* 2021;2(3):100141.
24. Maglott D, Ostell J, Pruitt KD, and Tatusova T. Entrez Gene: gene-centered information at NCBI. *Nucleic Acids Res.* 2011;39(Database issue):D52-7.

25. Wijesooriya K, Jadaan SA, Perera KL, Kaur T, and Ziemann M. Urgent need for consistent standards in functional enrichment analysis. *PLoS Comput Biol.* 2022;18(3):e1009935.
