# Supplementary material for: Multiomics reveals multilevel control of renal and systemic metabolism by the renal tubular circadian clock
Source: J Clin Invest. 2023 Apr 17;133(8):e167133. doi: 10.1172/JCI167133 (PMC10104904; doi:10.1172/JCI167133)
Supplement: Supplemental table 7 [file jci-133-167133-s082.pdf]

SUPPLEMENTARY TABLE 7. KEY RESOURCES TABLE

REAGENT or RESOURCE	SOURCE	IDENTIFIER
**Experimental models : Organisms/Strains**		
C57Bl/6	Charles River	027
B6.129S4(Cg)-Arntltm1Weit/J (Bmal1$^{flox/flox}$)	The Jackson Laboratory	#:007668
LC1-Cre	(1)	N/A
Pax8-rtTA	(1)	N/A
Bmal1$^{flox/flox}$ LC1-Cre Pax8-rtTA	This paper	
**Reagents**		
Phosphate Buffer 10X pH 7.4	Gibco	70011-044
Doxycycline Hyclate	Applichem	A2951
Standard diet	Kliba-Nafag	3242
**Data**		
RNA-seq data	This paper	GSE216252
Proteomics data	This paper	PXD036803 (Proteomexchange.org)
Metabolomics data	This paper	doi: 10.5281/zenodo.7225427
**Softwares and Algorithms**		
GraphPad Prism	GraphPad Software, LCC	v9.4.0
Xcalibur	ThermoFisher Scientific	v4.2
MaxQuant	Max-Planck-Institute of Biochemistry	v.1.6.14.0
Perseus	(2)	1.6.5.0
IMOD	Electron microscopy facility University of Lausanne	v4.10.35
bcl2fastq2	Illumina, Inc.	v2.20
Cutadapt	https://doi.org/10.14806/ej.17.1.200	v1.8
fastq_screen	(3)	v0.11.1
reaper	(3)	v15-065
STAR	(4)	v2.5.3a

htseq-count	(4)	v0.9.1
R	https://www.r-project.org/	v4.0.3, v4.1.0
Bioconductor (R packages for bioinformatics)	https://bioconductor.org/, (5)	v3.12, v3.13
dryR	(6) https://github.com/naef-lab/dryR	v1.0.0
edgeR	(7) https://doi.org/10.18129/B9.bioc.edgeR	
STAR	(8) https://code.google.com/archive/p/rna-star/	v. 2.5.3a

1. Traykova-Brauch M, Schonig K, Greiner O, Miloud T, Jauch A, Bode M, et al. An efficient and versatile system for acute and chronic modulation of renal tubular function in transgenic mice. *Nat Med.* 2008;14(9):979-84.
2. Tyanova S, Temu T, Sinitcyn P, Carlson A, Hein MY, Geiger T, et al. The Perseus computational platform for comprehensive analysis of (prote)omics data. *Nat Methods.* 2016;13(9):731-40.
3. Wingett SW, and Andrews S. FastQ Screen: A tool for multi-genome mapping and quality control. *F1000Res.* 2018;7:1338.
4. Davis MP, van Dongen S, Abreu-Goodger C, Bartonicek N, and Enright AJ. Kraken: a set of tools for quality control and analysis of high-throughput sequence data. *Methods (San Diego, Calif).* 2013;63(1):41-9.
5. Huber W, Carey VJ, Gentleman R, Anders S, Carlson M, Carvalho BS, et al. Orchestrating high-throughput genomic analysis with Bioconductor. *Nat Methods.* 2015;12(2):115-21.
6. Weger BD, Gobet C, David FPA, Atger F, Martin E, Phillips NE, et al. Systematic analysis of differential rhythmic liver gene expression mediated by the circadian clock and feeding rhythms. *Proc Natl Acad Sci U S A.* 2021;118(3).
7. Robinson MD, McCarthy DJ, and Smyth GK. edgeR: a Bioconductor package for differential expression analysis of digital gene expression data. *Bioinformatics.* 2010;26(1):139-40.
8. Dobin A, Davis CA, Schlesinger F, Drenkow J, Zaleski C, Jha S, et al. STAR: ultrafast universal RNA-seq aligner. *Bioinformatics.* 2013;29(1):15-21.
